# Comparative Analysis of T-Cell Spatial Proteomics and the Influence of HIV Expression

**DOI:** 10.1016/j.mcpro.2022.100194

**Published:** 2022-01-08

**Authors:** Aaron L. Oom, Charlotte A. Stoneham, Mary K. Lewinski, Alicia Richards, Jacob M. Wozniak, Km Shams-Ud-Doha, David J. Gonzalez, Nevan J. Krogan, John Guatelli

**Affiliations:** 1 Biomedical Sciences Doctoral Program, University of California San Diego, La Jolla, California, USA; 2 School of Medicine, University of California San Diego, La Jolla, California, USA; 3 Veterans Medical Research Foundation, La Jolla, California, USA; 4 VA San Diego Healthcare System, La Jolla, California, USA; 5 Proteomics Facility, Sanford Burnham Prebys Medical Discovery Institute, La Jolla, California, USA; 6 Department of Cellular and Molecular Pharmacology, University of California, San Francisco, California, USA; 7 Quantitative Biosciences Institute (QBI), University of California, San Francisco, California, USA; 8 Institute for Virology and Immunology, J. David Gladstone Institutes, San Francisco, California, USA; 9 Gladstone Institute of Data Science and Biotechnology, J. David Gladstone Institutes, San Francisco, California, USA; 10 Department of Pharmacology, University of California San Diego, La Jolla, California, USA; 11 Skaggs School of Pharmacy and Pharmaceutical Sciences, University of California San Diego, La Jolla, California, USA

**Keywords:** mass spectrometry, subcellular fractionation, HIV, spatial proteomics, Jurkat T cells, computational biology, differential centrifugation, inducible HIV, ACN, acetonitrile, AGC, automatic gain control, AP-MS, affinity purification-MS, BANDLE, Bayesian analysis of differential localization experiments, CCR5, chemokine receptor 5, DOM, Dynamic Organellar Mapping, ER, endoplasmic reticulum, FA, formic acid, FT, Fourier transform, HCMV, human cytomegalovirus, HPA, Human Protein Atlas, HRP, horseradish peroxidase, IT, injection time, MS, mass spectrometry, NIH, National Institutes of Health, PCA, principal component analysis, SVM, support vector machine, TAGM-MAP, t-augmented Gaussian mixture modeling with *maximum a posteriori* estimates, TMT, tandem mass tag, TRANSPIRE, translocation analysis of spatial proteomics, UCSD, University of California San Diego

## Abstract

As systems biology approaches to virology have become more tractable, highly studied viruses such as HIV can now be analyzed in new unbiased ways, including spatial proteomics. We employed here a differential centrifugation protocol to fractionate Jurkat T cells for proteomic analysis by mass spectrometry; these cells contain inducible HIV-1 genomes, enabling us to look for changes in the spatial proteome induced by viral gene expression. Using these proteomics data, we evaluated the merits of several reported machine learning pipelines for classification of the spatial proteome and identification of protein translocations. From these analyses, we found that classifier performance in this system was organelle dependent, with Bayesian t-augmented Gaussian mixture modeling outperforming support vector machine learning for mitochondrial and endoplasmic reticulum proteins but underperforming on cytosolic, nuclear, and plasma membrane proteins by QSep analysis. We also observed a generally higher performance for protein translocation identification using a Bayesian model, Bayesian analysis of differential localization experiments, on row-normalized data. Comparative Bayesian analysis of differential localization experiment analysis of cells induced to express the WT viral genome *versus* cells induced to express a genome unable to express the accessory protein Nef identified known Nef-dependent interactors such as T-cell receptor signaling components and coatomer complex. Finally, we found that support vector machine classification showed higher consistency and was less sensitive to HIV-dependent noise. These findings illustrate important considerations for studies of the spatial proteome following viral infection or viral gene expression and provide a reference for future studies of HIV-gene-dropout viruses.

## Highlights

• T cells with inducible HIV-1 genomes are a scalable system for spatial proteomics.
• HIV-1 expression minimally affected the consistency of protein classification.
• SVM classified proteins more consistently than TAGM-MAP.
• SVM and TAGM-MAP classifiers each have accuracy advantages for distinct organelles.
• BANDLE pipeline showed highest agreement with known HIV host interactors and targets.

## Graphical Abstract

**Figure:**
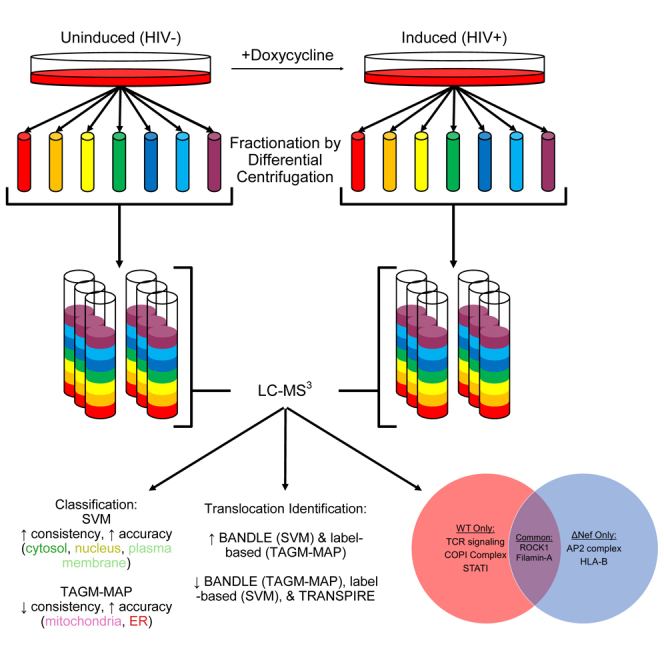

## In Brief

Using HIV-1 as a model virus, we compared several published analytical tools for spatial proteomics to determine the effect of viral expression. We found that when using differential centrifugation to fractionate T cells, the accuracy of classifiers was organelle dependent with variable sensitivity to viral gene expression. Identification of protein translocations by the BANDLE pipeline showed the highest agreement with known HIV interactors and targets. These findings lay a foundation for future spatial proteomics studies of viral infection and expression.

Spatial proteomics is a methodologically diverse and rapidly growing field within mass spectrometry (MS) that aims to understand the subcellular localization of the human proteome (1, 2, 3, 4, 5, 6, 7). While initial efforts focused on establishing techniques and reference maps for various cell lines, recent work by Cristea *et al.* (7) expanded the field to understand the whole-cell effects of viral infection using human cytomegalovirus (HCMV) as a prototype. This work led to novel findings on the importance of peroxisomes in herpesvirus infectivity (8), exemplifying the power of these methods for uncovering new viral biology. However, as this was a first in its class study, how different methodologies might impact the results of viral studies using spatial proteomics is unclear. Using the well-characterized HIV-1 as a model virus system, we aimed to compare the output of several published spatial proteomic analysis pipelines (9, 10, 11, 12) as a survey of established methods.

To model HIV expression, we used a Jurkat T-cell line that harbors a doxycycline-regulated HIV-1 genome. These cells were previously developed by our group to generate nearly homogenous HIV-positive cell populations for MS analysis (13). As an additional biological comparator, we examined both WT virus and a virus lacking the accessory gene *nef* (ΔNef). Nef is a small (27 kDa) myristoylated membrane–associated accessory protein expressed early during the viral replication cycle (14, 15). Nef increases viral growth rate and infectivity (16), and it dysregulates the trafficking of cellular membrane proteins such as CD4, class I major histocompatibility complex, and proteins involved in T-cell activation such as CD28 (17) and p56-Lck (18). Some of these activities enable the virus to evade immune detection (19, 20). Here, we use inducible Jurkat T-cell lines containing either WT or ΔNef HIV-1_NL4-3_ provirus and compare the spatial proteome of uninduced cells to cells postinduction with doxycycline. To fractionate the cells, we used a modified version of the Dynamic Organellar Mapping (DOM) protocol (5, 6) with additional centrifugation steps (4) to enhance organellar resolution and then analyzed the fractions by MS using tandem mass tag (TMT) multiplexing.

Following the generation and processing of MS data, two broad steps are required for spatial proteomics: classification and translocation event determination. For classifying detected proteins into cellular organelles, we compared two methods from pRoloc, an R software package developed by the Lilley laboratory (12). The first was support vector machine (SVM) classification, which outputs a label for each protein and an algorithm-specific confidence score that can be used to threshold assignments (1). The second was a Bayesian approach called t-augmented Gaussian mixture modeling with *maximum a posteriori* estimates (TAGM-MAP), which outputs a label for each protein and an actual probability of assignment (11). TAGM-MAP is one of two TAGM methods: TAGM with Markov-chain Monte Carlo is an alternative that yields full posterior probability distributions as opposed to the point estimates of localization probability yielded by TAGM-MAP (21). To gauge the quality of these classifications, we compared the two methods using the QSep metric developed by Lilley *et al.* (22), which quantifies the separation, or resolution, of the organelles in question. We in addition crossreferenced our organellar assignments to existing organellar proteome databases (23, 24, 25, 26).

After classification, data were analyzed for translocating proteins following HIV expression. We compared three different methods for determining protein translocations: label-based movement, translocation analysis of spatial proteomics (TRANSPIRE) (9), and Bayesian analysis of differential localization experiments (BANDLE) (10). Label-based movement relies strictly on identifying proteins that are consistently classified in one organelle prior to a cellular perturbation and then consistently classified in another organelle following the perturbation; this method was employed by Cristea *et al.* (7) in their HCMV study. TRANSPIRE is a refined methodology from the Cristea laboratory that relies on generating synthetic translocations from proteins of known localization and uses Gaussian process analysis to determine the likelihood of proteins of unknown localization behaving in a manner consistent with anticipated translocations following a cellular perturbation (9). Finally, BANDLE is another method developed by Lilley *et al.* (10) that takes replicated data, both with and without a perturbation, and uses Bayesian analysis to yield a ranked list of possible translocations with their associated likelihood of occurrence. We compared the translocation events from these various methods by crossreferencing events with a previous study of the HIV interactome (27) as well as the more broad National Institutes of Health (NIH) HIV-1 Human Interaction Database (28).

From these comparisons, we found that the performance of different classifiers is organelle dependent and shows varied effects from HIV expression. As determined by agreement with previously published organellar proteomes, classification with TAGM-MAP showed increased accuracy in mitochondrial and endoplasmic reticulum (ER)–classified proteins, whereas SVM outperformed TAGM-MAP with nuclear, cytosolic, and plasma membrane–classified proteins. We also observed generally higher performance for protein translocation using BANDLE on row-normalized data (*i.e.*, normalization used for SVM classification) when compared with the HIV interactomes. BANDLE analysis of WT and ΔNef data identified known Nef interactors involved in T-cell activation and the coatomer complex. The need to combine similarly behaving organelles for TRANSPIRE analysis hampered the performance of that method. Finally, we found that SVM classification showed higher consistency and was less sensitive to HIV-dependent noise. These findings illustrate the complexities in choosing a computational method for spatial proteomics study and serve as a foundation for additional studies.

## Experimental Procedures

### Experimental Design and Statistical Rationale

All fractionation experiments with mass spectrometric analysis were performed in technical triplicate for each condition (uninduced and induced), with two biological replicates for WT and ΔNef NL4-3 Jurkat cells. This yielded a total of six uninduced and six induced technical replicates for each virus type. Biological replicates were prepared on separate days and analyzed by MS on separate days. Western blotting and flow cytometry were performed on each technical replicate. Analyses for QSep used Welch's *t* test to determine statistical significance.

### Cell Culture

The doxycycline-inducible NL4-3 HIV-1 and NL4-3 ΔNef Jurkat cell lines were previously described (13, 29). The replication-incompetent genome used was based on pNL4-3 but lacked most of the 5′ U3 region, encoded a self-inactivation deletion in the 3′ long terminal repeat, and contained the V3 region from the R5-tropic 51-9 virus (30) to prevent the cell–cell fusion of the Jurkat T cells used herein, which do not express chemokine receptor 5 (CCR5). Inducible cells were cultured in RPMI1640 media supplemented with penicillin/streptomycin and 10% Tet-free fetal bovine serum, as well as puromycin (1 μg/ml) and G418 (200 μg/ml) to maintain persistence of the tetracycline transactivator and the inducible genome. Cells were passaged every 2 days to keep concentrations between 3.5 × 10^5^ and 1 × 10^6^ cells/ml. Cells were maintained at 37°C, 5% CO_2_, and 95% humidity.

### Doxycycline Induction and Fractionation

On the day before fractionation, 2.016 × 10^9^ cells were plated at 6 × 10^5^ cells/ml in T75 flasks at a total volume of 40 ml/flask. Half of these cells were induced to express HIV-1/HIV-1ΔNef with doxycycline (1 μg/ml) for 18 h, whereas the other half remained uninduced. Following induction, cells of each condition, that is, uninduced and induced, were split into three technical replicates and then centrifuged at 500*g* for 5 min at 4°C. Each technical replicate was pooled into a single 50 ml tube using ice-cold 1× PBS and then counted by hemocytometer. From each technical replicate, 3 × 10^8^ cells were fractionated. Two aliquots of cells were taken from each technical replicate for whole-cell Western blots and testing induction by flow cytometry.

The fractionation protocol used here is derived from the Dynamic Organellar Maps method (5) with additional centrifugation steps (4) and TMT-based MS analysis rather than stable isotope labeling by/with amino acids in cell culture (6). Cells for fractionation were centrifuged at 500*g* for 5 min at 4°C, then resuspended in ice-cold PBS, and incubated for 5 min on ice. Cells were again centrifuged at 500*g* for 5 min at 4°C, then resuspended in ice-cold hypotonic lysis buffer (25 mM Tris–HCl [pH 7.5], 50 mM sucrose, 0.5 mM MgCl_2_, and 0.2 mM EGTA in water), and incubated for 5 min on ice. Using a 7 ml Dounce homogenizer, cells were homogenized with 20 full strokes of the tight pestle. Cell homogenates were then immediately transferred to a 13 ml (14 × 89 mm) ultracentrifuge tube with sufficient ice-cold hypertonic sucrose buffer (1.25 M sucrose, 25 mM Tris–HCl [pH 7.5], 0.5 mM MgCl_2_, and 0.2 mM EGTA in water) to restore 250 mM sucrose concentration. All replicates were then centrifuged at 1000*g* for 10 min at 4°C in a Beckman Coulter ultracentrifuge (SW-41 Ti rotor), balancing each tube with balance buffer (250 mM sucrose, 25 mM Tris–HCl [pH 7.5], 0.5 mM MgCl_2_, and 0.2 mM EGTA in water). Supernatants were transferred to a fresh ultracentrifuge tube, balanced with balance buffer, and then fractionated using the following differential centrifugation protocol: 3000*g* for 10 min, 5400*g* for 15 min, 12,200*g* for 20 min, 24,000*g* for 20 min, 78,400*g* for 30 min, 110,000*g* for 35 min, and 195,500*g* for 40 min. All centrifugation steps were performed at 4°C with pellets from each spin being resuspended in SDS buffer (2.5% SDS and 50 mM Tris–HCl [pH 8.0] in water). Fractions were then heated for 10 min at 72°C. Protein content of each fraction was quantified in triplicate using a bicinchoninic acid protein assay (Thermo Fisher Scientific).

### Confirmatory Western Blots and p24 Flow Cytometry

Prior to mass spectrometric analysis of fractions, induction and fractionation were evaluated by flow cytometry and Western blotting. For p24 flow cytometry, an aliquot of 2 × 10^6^ cells from each technical replicate was pelleted at 500*g* for 5 min at 4°C and then resuspended in ice-cold fluorescence-assisted cell sorting buffer (2% fetal bovine serum and 0.1% sodium azide in 1× PBS). The cells were again pelleted at 500*g* for 5 min at 4°C, then resuspended in Cytofix/Cytoperm reagent (BD Biosciences), and incubated on ice for 30 min Following fixation/permeabilization, cell suspensions were diluted with wash buffer and pelleted at 500*g* for 5 min at 4°C. Cells were resuspended in p24 primary antibody solution (1:100 dilution of p24-FITC antibody clone KC57 [Beckman Coulter] diluted in perm/wash buffer) and incubated on ice for 30 min in darkness. Ice-cold fluorescence-assisted cell sorting buffer was added to each sample, and cells were pelleted at 500*g* for 5 min at 4°C. The intracellular p24 was analyzed using an Accuri C6 flow cytometer (BD Biosciences). Uninduced cells had an average p24+ population of 0.27% (SD = 0.20) and live cell population of 85.78% (SD = 3.37). Induced cells had an average p24+ population of 94.85% (SD = 1.23) and live cell population of 79.25% (SD = 4.35).

An aliquot of 1 × 10^7^ cells from each technical replicate was lysed in SDS buffer and probe sonicated on ice until no longer viscous. 3000*g* fractions were also probe sonicated. The samples were mixed with 4× loading buffer (200 mM Tris–HCl [pH 6.8], 8% SDS, 40% glycerol, 200 mM Tris(2-carboxyethyl)phosphine-HCl, and 0.04% bromophenol blue in water), and proteins were then separated on 10% SDS-PAGE gels at a constant 70 V. Proteins were transferred to polyvinylidene difluoride membranes for 1 h using the Trans-Blot turbo (Bio-Rad) system using standard conditions. The membranes were blocked in 5% milk in 1× PBS + 0.1% Tween-20 for 30 min at room temperature prior to incubation with primary antibodies diluted in 1% milk and 0.05% sodium azide in 1× PBS + 0.1% Tween-20: sheep anti-Nef (gift from Celsa Spina; diluted 1:3000), mouse anti-p24 (Millipore; diluted 1:500), Chessie8 (mouse anti-gp41; NIH AIDS Research and Reference Reagent program (31); diluted 1:10,000), rabbit anti-Vpu (NIH AIDS Research and Reference Reagent program ARP-969; contributed by Dr Klaus Strebel; diluted 1:1000), and mouse anti-GAPDH (GeneTex; diluted 1:5000). The blots were washed and probed with either horseradish peroxidase (HRP)–conjugated goat antimouse, HRP-goat anti-rabbit, or HRP-rabbit antisheep secondary (Bio-Rad) diluted 1:3000, incubating for 1 h at room temperature on a shaker. Apparent molecular mass was estimated with PageRuler protein standard (Thermo Fisher Scientific). Blots were imaged using Western Clarity detection reagent (Bio-Rad) before detection on a Bio-Rad Chemi Doc imaging system with Bio-Rad Image Lab, version 5.1 software.

### Sample Digestion for MS

Disulfide bonds were reduced with 5 mM Tris(2-carboxyethyl)phosphine-HCl at 30°C for 60 min, and cysteines were subsequently alkylated (carbamidomethylated) with 15 mM iodoacetamide in the dark at room temperature for 30 min. Proteins were then precipitated with nine volumes of methanol, pelleted, and resuspended in 1 M urea and 50 mM ammonium bicarbonate. Following precipitation, protein concentration was determined using a bicinchoninic acid protein assay. A total of 0.2 mg of protein was subjected to overnight digestion with 8.0 μg of mass spec grade Trypsin/Lys-C mix (Promega). Following digestion, samples were acidified with formic acid (FA), and subsequently, 150 μg peptides were desalted using AssayMap C18 cartridges mounted on an Agilent AssayMap BRAVO liquid handling system. C18 cartridges were first conditioned with 100% acetonitrile (ACN), followed by 0.1% FA. The samples were then loaded onto the conditioned C18 cartridge, washed with 0.1% FA, and eluted with 60% MeCN and 0.1% FA. Finally, the organic solvent was removed in a SpeedVac concentrator prior to LC–MS/MS analysis.

### TMT Labeling

Peptide concentration was determined using a Nanodrop, and a total of 15 μg of peptide was then used for TMT labeling, each replicate serving as a multiplex. Briefly, dried peptide sample was resuspended in 200 mM Hepes (pH 8) and incubated for 1 h at room temperature with one of the TMT10-plex reagents (Thermo Fisher Scientific) solubilized in 100% anhydrous ACN. Reactions were quenched using a 5% hydroxylamine solution at 1 to 2 μl per 20 μl TMT reagent. The multiplexed samples were then pooled and dried in a SpeedVac. The labeled peptides were resuspended in 0.1% FA. After sonication for 1 min, the sample was desalted manually using SepPak; the column was first conditioned with 100% ACN, followed by 0.1% FA. Sample was loaded, then washed with 0.1% FA, and eluted in a new vial with 60% ACN and 0.1% FA. Finally, the organic solvent was removed using a SpeedVac concentrator prior to fractionation.

### High pH Reverse-Phase Fractionation

Dried samples were reconstituted in 20 mM ammonium formate (pH ∼10) and fractionated using a Waters ACQUITY CSH C18 1.7 μm 2.1 × 150 mm column mounted on a MClass Ultra Performance Liquid Chromatography system (Waters Corp) at a flow rate of 40 μl/min with buffer A (20 mM ammonium formate [pH 10]) and buffer B (100% ACN). Absorbance values at 215 and 280 nm were measured on a Waters UV–Vis spectrophotometer, using a flowcell with a 10 mm path length. Peptides were separated by a linear gradient from 5% B to 25% B in 62.5 min followed by a linear increase to 60% B in 4.5 min and 70% in 3 min and maintained for 7 min before increasing to 5% in 1 min. Twenty-four fractions were collected and pooled in a noncontiguous manner into 12 total fractions. Pooled fractions were dried to completeness in a SpeedVac concentrator.

### LC–MS^3^ Analysis

Dried samples were reconstituted with 0.1% FA and analyzed by LC–MS/MS on an Orbitrap Fusion Lumos mass spectrometer (Thermo Fisher Scientific) equipped with an Easy nLC 1200 ultra-high pressure liquid chromatography system interfaced *via* a Nanospray Flex nanoelectrospray source (Thermo Fisher Scientific). Samples were injected on a C18 reverse phase column (25 cm × 75 μm packed with Waters BEH 1.7 μm particles) and separated over a 120-min linear gradient of 2 to 28% solvent B at a flow rate of 300 nl/min. The mass spectrometer was operated in positive data-dependent acquisition mode.

Parameter settings were set as follows: Fourier transform (FT) MS1 resolution (120,000) with automatic gain control (AGC) target of 1e6, injection time (IT) MS2 isolation window (0.4 *m/z*), IT MS2 maximum IT (120 ms), IT MS2 AGC (2E4), IT MS2 collision-induced dissociation energy (35%), synchronous precursor selection ion count (up to 10), FT MS3 isolation window (0.4 *m/z*), FT MS3 maximum IT (150 ms), and FT MS3 resolution (50,000) with AGC target of 1e5. A TOP10 method was used where each FT MS1 scan was used to select up to 10 precursors for interrogation by collision-induced dissociation MS2 with readout in the ion trap. Each MS2 was used to select precursors (synchronous precursor selection ions) for the MS3 scan, which measured reporter ion abundance.

Subset analyzed using an MS2 method, with an MS1 Orbitrap resolution of 120,000, MS1 scan range of 350 to 1400 *m/z*, MS1 AGC of 1e6, and an MS1 maximum IT of 100 ms. Cycle time was set to 3 s. Quadrupole isolation window was set to 1.6. MS2 spectra were analyzed by higher-energy collision-induced dissociation at a collision energy of 35 and an Orbitrap resolution of 50,000. Maximum IT was set to 86 ms, with an MS2 AGC target of 1.5e5.

### MS Spectra Identification

Raw files were analyzed using Proteome Discoverer, version 2.3 (Thermo Fisher Scientific). MS/MS spectra were searched against a concatenated database containing UniProt human and HIV-1 proteins (downloaded February 3, 2020) and reverse decoy sequences using the Sequest algorithm (32); the database contained 20,367 total entries. Mass tolerance was specified at 50 ppm for precursor ions and 0.6 Da for MS/MS fragments. Static modifications of TMT 10-plex tags on lysine and peptide n-termini (+229.162932 Da) and carbamidomethylation of cysteines (+57.02146 Da) and variable oxidation of methionine (+15.99492 Da) were specified in the search parameters. Data were filtered to a 1% false discovery rate at the peptide and protein levels through Percolator (33) using the target-decoy strategy (34). TMT reporter ion intensities were extracted from MS3 spectra within Proteome Discoverer to perform quantitative analysis.

### Computational Analysis

Matching biological replicates were combined (*i.e.*, WT biological replicate 1 and 2) and then analyzed using the various pipelines described. The *Homo sapiens* (“hsap”) marker set from pRoloc was used in all cases. For classification and translocation event identification, only the proteins commonly detected across matched biological replicates were analyzed to allow for consistency in comparing methods on the same dataset.

The pRoloc implementation of SVM (12) was performed on row-normalized datasets, whereas the pRoloc implementation of TAGM-MAP (11) required principal component analysis (PCA) transformation and no row normalization with the first four principal components carried forward. The PCA transformation was used because of floating point arithmetic errors that arose because of highly correlated features. Default parameters for algorithms were used excepting the following:

SVM hyperparameter classification: 10 times 10-fold crossvalidation

SVM classification threshold: median algorithm score for each organelle

TAGM-MAP model training: 200 iterations

BANDLE: six chains

TRANSPIRE was run on averaged row-normalized datasets, that is, technical replicates were row normalized and then values for each feature were averaged for each protein across matched technical replicates. Organelles were combined into five groups: (1) Golgi apparatus/plasma membrane/ER/peroxisomes/lysosomes, (2) cytosol/actin cytoskeleton/proteasome, (3) nucleus, (4) mitochondria, and (5) 40S/60S ribosome. The number of inducing points and the kernel function were chosen from amongst the suggested values in the TRANSPIRE documentation. For these datasets, 75 inducing points and the squared exponential kernel performed best and were used in the analysis.

The average distribution of proteins across organelles was calculated by determining the average organellar distribution for a single technical replicate and then averaging the values of matched technical replicates. Marker profiles were generated by averaging the behavior of markers for a given organelle within a technical replicate and then averaging those values across technical replicates for each organelle. Organellar QSep scores were calculated by averaging the individual QSep scores between two organelles across all matched technical replicates and then plotting the distribution of those averages.

Comparisons to the Human Protein Atlas (HPA) were completed by combining several HPA subcellular localization annotations to align with the organelles used by pRoloc:

1. Nuclear membrane, nucleoli fibrillar center, nucleoli rim, nucleoli, kinetochore, mitotic chromosome, nuclear bodies, nuclear speckles, and nucleoplasm: Nucleus
2. Actin filaments and focal adhesion sites: Actin cytoskeleton
3. Plasma membrane and cell junctions: Plasma membrane

Remaining designations within the HPA beyond the aforementioned and those in common with pRoloc's “hsap” markers were not considered. The 40S ribosome, 60S ribosome, and proteasome classes from the SVM and TAGM-MAP classified data were collapsed into the cytosol label.

Thresholds for expected number of protein translocations were determined by dividing the size of the Jӓger HIV interactome (27), 453 proteins, or the NIH HIV interactome (28), 4628 proteins, by the predicted human proteome size of 19,773 proteins (35). Gene Ontology analysis was conducted using the STRING database (36).

## Results

### Doxycycline-Inducible HIV-1_NL4-3_ Jurkat T Cells are a Scalable and Uniform System for Subcellular Fractionation and Proteomic Studies

**Fig. 1:**
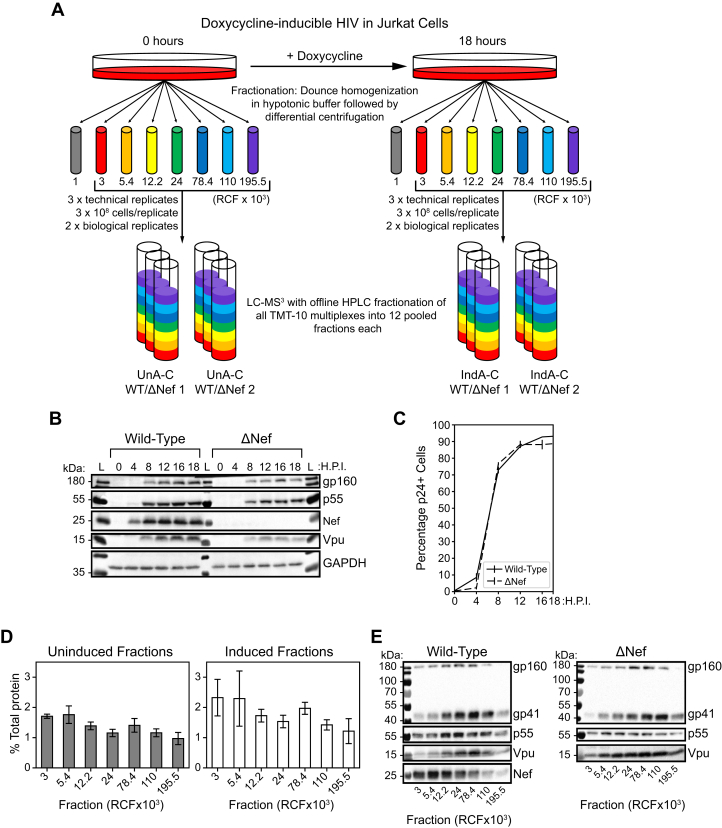
**Inducible HIV-1 Jurkat cell lines yield a near pure population of HIV-expressing cells suitable for fractionation by differential centrifugation.***A*, equal numbers of doxycycline-inducible WT and ΔNef HIV Jurkat cells were induced or left uninduced for 18 h and then fractionated by Dounce homogenization in a hypotonic lysis buffer. Cell homogenates were put through a differential centrifugation protocol, discarding the nuclear pellet (1000*g*) and lysing remaining pellets in 2.5% SDS buffer. Fractions were labeled for TMT-10 multiplexing and further offline HPLC fractionation. All multiplexes were run for 3 h on LC–MS^3^. *B*, Western blot showing induction of HIV p55, gp160, gp41, Nef, and Vpu with a GAPDH loading control. Cells were induced for 0, 4, 8, 12, 16, and 18 h, lysed, and then a portion of these cell lysates was run on 10% SDS-PAGE gels. *C*, flow cytometry analysis of remaining sample from *B*. HIV-1 expression peaked at ∼95% of cells p24+ by 18 h. *D*, average percentage of total cellular protein detected in each fraction by BCA protein assay. Bars represent the mean value for a given fraction based on the average from each biological replicate. Error bars are one standard deviation. All BCA assays were performed in technical triplicate on 10-fold dilutions for each biological replicate. *E*, Western blots for cell fractions of inducible WT HIV Jurkat cells (*left*) and ΔNef HIV Jurkat cells (*right*), 18 h postinduction. Blots shown are representative of both biological replicates. BCA, bicinchoninic acid; TMT, tandem mass tag.

The WT HIV-1-inducible cells used here were previously generated and used for whole-cell quantitative proteomics and phosphoproteomics (13). To avoid the formation of syncytia, which could alter the subcellular fractionation and subsequent spatial proteomic data, the inducible HIV-1_NL4-3_ genomes were modified with a CCR5-tropic Env protein to avoid cell–cell fusion between the CCR5-negative Jurkat cells. Because of the high induction rates of HIV-1 expression and the scalability of this culture system, we reasoned that it would be amenable to subcellular fractionation by differential centrifugation with subsequent MS analysis (Fig. 1*A*). To determine the optimal time point for analysis following induction of HIV-1 expression, cells were treated with doxycycline for 0, 4, 8, 12, 16, and 18 h, and the expression of HIV-1 proteins was detected by Western blotting and flow cytometry (Fig. 1, *B* and *C*). WT cells began to express detectable Nef by 4 h postinduction, and both WT and ΔNef cells expressed p55 Gag precursor (the precursor protein for virion structural proteins) by 8 h and gp160 (the envelope glycoprotein precursor) by 12 h. By 18 h, viral proteins were robustly expressed; about 90 to 95% of both WT and ΔNef cells were positive by flow cytometry for p24 capsid (a proteolytic product of p55).

Subcellular fractionation was performed 18 h postinduction; the cells were mechanically ruptured with a Dounce homogenizer in hypotonic solution and then subjected to a differential centrifugation protocol before preparation for quantitative and multiplexed MS analysis. Uninduced and induced cells were handled in technical triplicate for each biological replicate (n = 2). We used a modified version of the DOM protocol (5, 6) with additional fractions generated at 110,000*g* and 195,500*g* to increase the resolution of the classification analysis; a similar method of expanded differential centrifugation fractionation was previously described by Lilley *et al.* (37). As a quality control before MS, protein yields were quantified for each fraction (Fig. 1*D*). The postnuclear fractions accounted for only ∼10 to 15% of total cellular protein, presumably because nuclear proteins and soluble cytoplasmic proteins that failed to pellet at 195,500*g* were discarded, leaving primarily membranous organelles or organellar fragments and large cytoplasmic complex proteins in the fractions analyzed. We also observed decreasing protein yields across the fractions, with an increase in the 78,400*g* fraction, consistent with the original DOM study using HeLa cells (5). In further support of differential fractionation, varied abundances of viral proteins across the fractions in cells expressing either the WT or ΔNef genomes were observed by Western blotting (Fig. 1*E*). Following confirmation of differential fractionation, we analyzed all fractions by LC–MS^3^ with TMT-10 multiplexing (Fig. 1*A*).

To determine the consistency of the MS analysis, we used unsupervised hierarchical clustering by Spearman correlation coefficient for the individual fractions. We found that for both the WT and ΔNef data, the fractions clustered by *g*-force rather than biological replicate (supplemental Figs. S1 and S2), suggesting consistent quantification values. Because the WT and ΔNef Jurkat cell lines represent individual clones for each, we also compared the uninduced fractions of the WT and ΔNef data to each other. This comparison showed that fractions still clustered by *g*-force rather than HIV genome (supplemental Fig. S3).

### SVM Yields Greater QSep Scores Than TAGM-MAP Even With Stringent Thresholds of Classification for TAGM-MAP

**Fig. 2:**
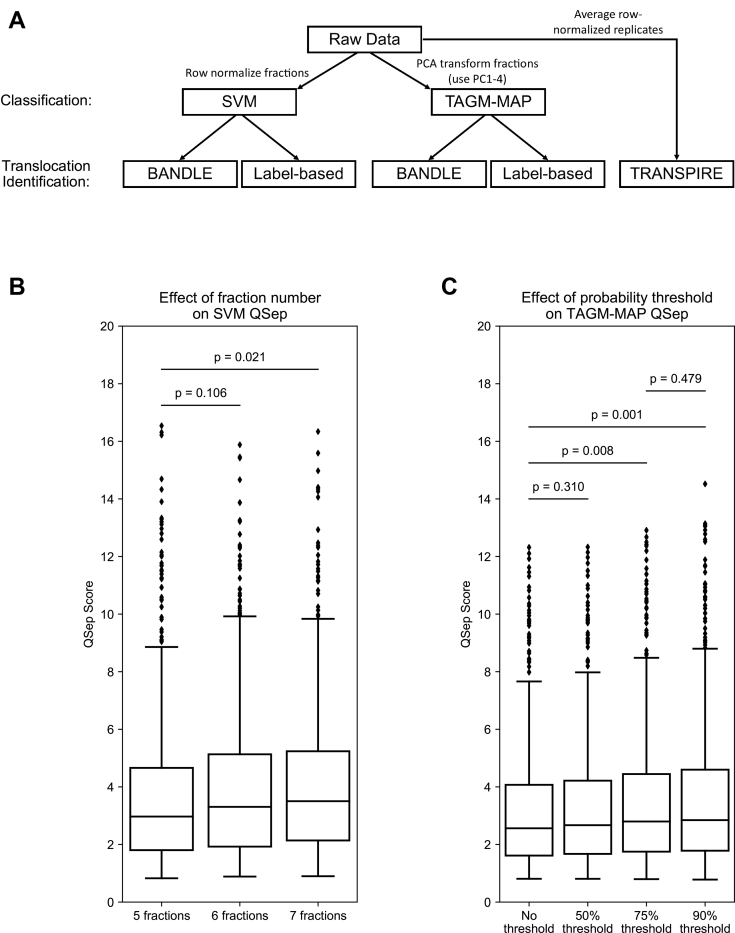
**Analysis of fractionation data reveals increased organellar resolution from added fractions and thresholding TAGM-MAP data.***A*, diagram of the computational methods used here. For SVM classification, the raw data of individual technical replicates were row normalized. For TAGM-MAP classification, the raw data of individual technical replicates were PCA transformed, with the first four principal components (PC1–4) carried forward for analysis. Both SVM and TAGM-MAP classified data were fed into BANDLE or label-based movement analysis. Finally, for analysis with TRANSPIRE, individual technical replicates were row normalized and then averaged together. *B*, boxplot of QSep scores for SVM analysis of WT uninduced samples using the original five fractions described by Itzhak *et al.* (5), adding a 110,000*g* fraction (six fractions), or adding both a 110,000*g* and a 195,500*g* fraction (seven fractions). *C*, boxplot of QSep scores for TAGM-MAP analysis of WT uninduced samples comparing using no threshold for remaining classified, a 50% chance of classification, a 75% chance of classification, or a 90% chance of classification. Statistical significance is calculated using a two-sided independent Student's *t* test with Welch's correction for unequal variance. Boxplots show median, not mean, line. BANDLE, Bayesian analysis of differential localization experiments; PCA, principal component analysis; SVM, support vector machine; TAGM-MAP, t-augmented Gaussian mixture modeling with *maximum a posteriori* estimates; TRANSPIRE, translocation analysis of spatial proteomics.

To classify the fractionation data and identify translocating proteins, we employed a variety of previously published methods (Fig. 2*A*). As several resources detail known HIV interactors (27, 28), we primarily focused on comparing classification and translocation identification methods using our WT data. In subsequent analyses, we examined the ΔNef data to determine the power of various methods in identifying Nef-specific effects.

Proteins were classified using either the pRoloc implementation of SVM or TAGM-MAP. As the differential centrifugation protocol employed here is a modified version of the DOM method, which generates only five fractions (5), we first examined whether our two additional fractions improved organellar resolution. The DOM method classifies proteins with SVM, so we compared the resolution of organelles with the QSep analysis (22) using the first five fractions for SVM classification, then the first six fractions, and finally all seven fractions (Fig. 2*B*). Notably, while QSep scores are often used to evaluate the distribution of marker proteins, here, we analyzed complete organellar clusters including both markers and newly classified proteins. As well-chosen marker proteins will tend toward the center of an organellar cluster, our use of QSep scores here offers only a partial picture of organellar resolution, with this depiction becoming murkier particularly toward the boundaries of each organelle. Taking this into consideration, we found that while the addition of the 110,000*g* spin alone had no significant effect on organellar resolution as compared with the original method, the subsequent addition of the 195,500*g* spin yielded a significant increase from a mean QSep score of 3.74 to 4.05 (median scores 2.97 and 3.50, respectively). In light of this, all subsequent analyses on the SVM data were performed on the full seven fractions. For these analyses, a simple median SVM score threshold was used for each organelle to determine which proteins remained classified or were designated unknown; this is explored further in the Discussion section.

To determine if an alternate method for classification would perform better than SVM, we also tested the pRoloc implementation of TAGM-MAP. TAGM-MAP has three primary outputs: an organellar localization, a probability that the given protein is located in that organelle, and a probability that a protein belongs to an outlier compartment. For our purposes, we focused on the localization and organellar probability, leaving the outlier probability aside. These localization probabilities allowed us to test the effect of different probability thresholds on QSep scores of TAGM-MAP. Using a 50% threshold, that is, converting all proteins with a probability of localization lower than 50% to an “unknown” designation, showed no significant effect, whereas both 75% and 90% thresholds showed significant gains over no thresholding (Fig. 2*C*). A 90% threshold showed no significant increase in QSep scores over the 75% threshold, so subsequent analyses employed the 75% threshold for TAGM-MAP classification. Of importance, we observed that the QSep scores from SVM classification were on average higher than those from TAGM-MAP even when comparing TAGM-MAP's highest condition (90% probability threshold, average score of 3.55) to SVM's lowest condition (five fractions, average score of 3.74). Notably, the probabilities of TAGM are better calibrated than the algorithm scores generated by SVM; the original TAGM publication shows a better correlation of classifier confidence with classifier accuracy for TAGM than SVM (21).

### SVM Classifies Proteins More Consistently Than TAGM-MAP

**Fig. 3:**
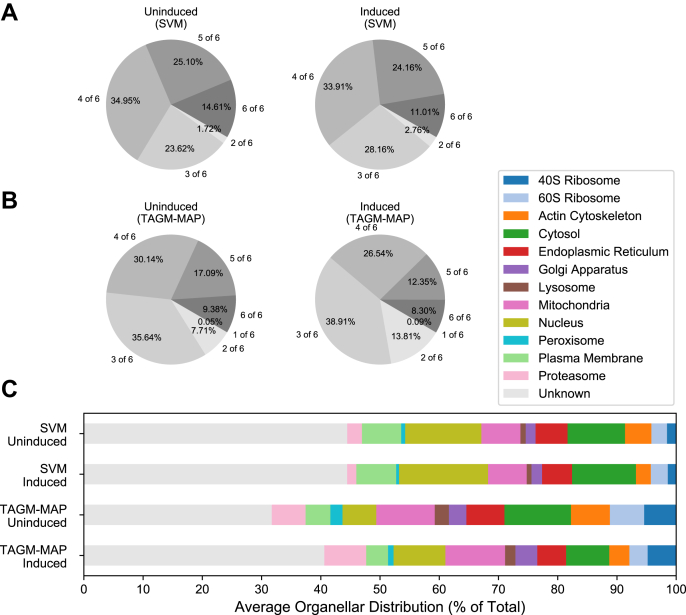
**Classification with SVM shows greater consistency than TAGM-MAP classification.***A*, proteins were classified by SVM, and the most frequent organellar classification was identified along with its frequency, that is, number of technical replicates classified as such. *Left pie chart* shows consistency of classification for WT uninduced replicates, and *right pie chart* shows WT induced replicates. *B*, same as (*A*), but classification by TAGM-MAP. *C*, average distribution of proteins across organelles for each indicated condition. All charts consider the same common proteins found across all WT replicates (4765 proteins). SVM, support vector machine; TAGM-MAP, t-augmented Gaussian mixture modeling with *maximum a posteriori* estimates.

We next wanted to understand how the SVM and TAGM-MAP methods compared for consistency of classification across WT replicates (Fig. 3, *A* and *B*). Using the median SVM score for each organelle to threshold SVM classifications, and the 75% organellar classification probability threshold for TAGM-MAP, both SVM (Fig. 3*A*) and TAGM-MAP (Fig. 3*B*) showed a low percentage (∼10–15%) of proteins that were classified identically in six of six technical replicates for either WT uninduced or induced. However, allowing for a majority of replicates, that is, four of six, gave ∼70 to 75% of proteins as classified consistently by SVM (Fig. 3*A*). This compared to ∼50 to 55% of proteins classified to a similar consistency by TAGM-MAP (Fig. 3*B*). HIV expression modestly decreased the consistency of both SVM and TAGM-MAP (∼5% difference), suggesting an increase in experimental noise from HIV expression. In addition, we found that SVM had a greater proportion of classified proteins that were consistently designated unknown as compared with TAGM-MAP for uninduced replicates (54 *versus* 35%), but this was not the case for induced replicates (56 *versus* 68%).

Looking at the average distribution of proteins across organelles, we found that SVM yielded a higher percentage of proteins that reverted to an unknown designation (Fig. 3*C*, 44% of proteins); this may partly explain the higher QSep scores generally seen for SVM compared with TAGM-MAP (Fig. 2). However, this percentage is stable between WT uninduced and induced replicates, whereas the lower percentage of unknown proteins (32% for uninduced and 41% for induced) for TAGM-MAP is more sensitive to HIV expression. This could explain the increase in consistently unknown proteins in the induced condition for TAGM-MAP relative to SVM. Similar trends were seen within the ΔNef data (supplemental Fig. S4). Marker behavior for WT (supplemental Fig. S5) and ΔNef (supplemental Fig. S6) is also similar, which likely explains the consistent trends. These data show a greater consistency for SVM classification, but with a relatively high proportion of proteins being designated unknown; the data in addition suggest that SVM is less susceptible to noise introduced by HIV expression.

### Agreement Between SVM and TAGM-MAP Classification Is Organelle Dependent and is Variably Affected by HIV Expression

**Fig. 4:**
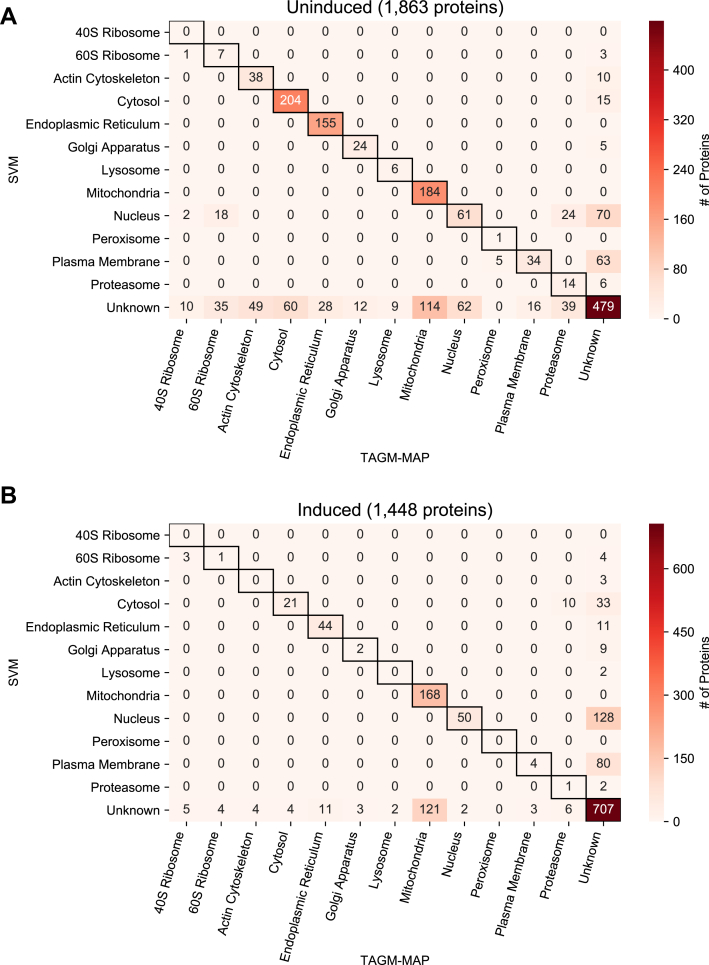
**Concordance of SVM and TAGM-MAP classifications depends on organelle and expression of HIV.***A*, heat map of common proteins that were consistently classified (proteins classified consistently in at least four of six replicates) by both SVM and TAGM-MAP for uninduced condition. Annotations indicate number of proteins in a given scenario. *B*, same as (*A*) for induced condition. SVM, support vector machine; TAGM-MAP, t-augmented Gaussian mixture modeling with *maximum a posteriori* estimates.

To determine the concordance of SVM and TAGM-MAP for classification, we examined all proteins that were classified consistently in at least four of six WT replicates for both SVM and TAGM-MAP. We found more such proteins for the uninduced replicates (Fig. 4*A*), 1863 proteins, as compared with the induced replicates (Fig. 4*B*) with 1448 proteins. This difference may be attributable to the decrease in classification consistency caused by HIV expression for both SVM and TAGM-MAP, which would be accentuated by any increased susceptibility of TAGM-MAP to HIV-dependent noise. Of these consistently classified proteins, HIV expression minimally affected classifier agreement; 65% agreed between SVM and TAGM-MAP for WT uninduced and 69% agreed between SVM and TAGM-MAP for induced replicates (see diagonal of heat maps). However, HIV expression increased the proportion of proteins that were consistently designated unknown by both SVM and TAGM-MAP: in uninduced cells, 40% of proteins agreed upon by the two methods were designated unknown (Fig. 4*A*), whereas 71% of agreed upon proteins were designated unknown from induced cells (Fig. 4*B*). This shift seems primarily driven by the increase in unknown designations for TAGM-MAP following HIV expression: in uninduced replicates, 52% of proteins designated unknown by SVM agreed with TAGM-MAP, but in induced replicates, 81% of these proteins agreed with TAGM-MAP. Matching trends were seen in ΔNef data (supplemental Fig. S7). Taken together, these data suggest that while HIV expression has little effect on the proportion of consistently classified proteins that are agreed upon by the two classifiers, the proportion of these proteins that are designated unknown is increased, and the overall number of consistently classified proteins is decreased.

We found that proteins from the cytosol, ER, and mitochondria were the most frequent among consistently classified proteins. These three organelles also showed the best agreement between SVM and TAGM-MAP for uninduced replicates (Fig. 4*A* and supplemental Fig. S4*A*). However, HIV expression decreased the proportion of cytosolic proteins and ER proteins in agreement between SVM and TAGM-MAP: 73% of all proteins classified as cytosolic and 85% of all proteins classified as ER agreed for WT uninduced replicates, but only 31% of cytosolic proteins and 67% of ER proteins agreed for induced replicates. This decrease was smaller for mitochondrial proteins: 62% for uninduced and 58% for induced. Similar trends for cytosolic and mitochondrial proteins were seen in ΔNef data, but ER proteins showed little change (supplemental Fig. S7). These data show an organelle-dependent trend in classifier agreement that is variably affected by HIV expression.

### TAGM-MAP Classification Yields Higher Agreement Than SVM Classification With Reported ER and Mitochondria Proteomes but Lower Agreement in Other Organelles

**Fig. 5:**
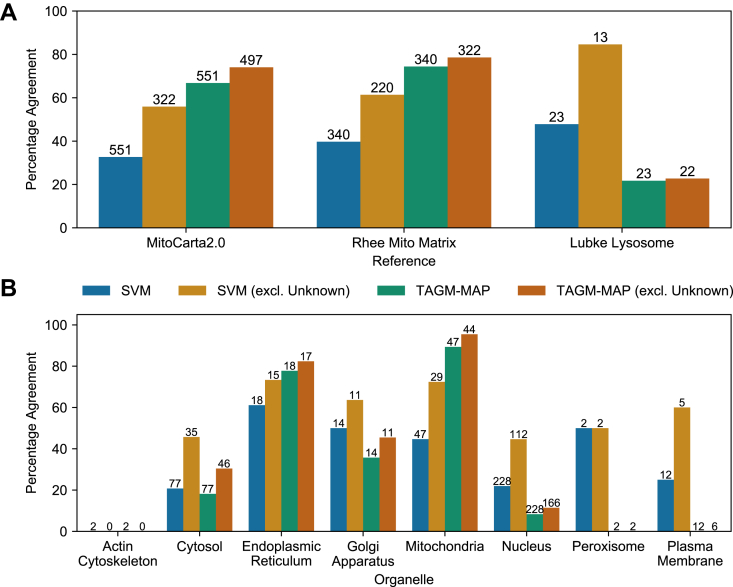
**Validation of protein classification reveals higher performance for ER and mitochondria using TAGM-MAP, but better performance for Golgi apparatus, nucleus, and plasma membrane using SVM.***A*, percentage of detected proteins from MitoCarta2.0 database (23), Rhee *et al.* (24) mitochondrial matrix study, or Lubke lysosome proteome (25) that were consistently classified (proteins classified consistently in at least four of six replicates) in line with the respective reference. Numbers above bars indicate the total number of proteins from that reference that were detected and classified for a given method. *B*, proteins classified by SVM or TAGM-MAP were crossreferenced against the HPA, and any protein considered to be singularly localized with an enhanced rating was kept. The percentage of these proteins that were consistently classified by SVM or TAGM-MAP into the HPA-designated organelle is shown. Numbers above bars indicate the number of HPA proteins considered for each organelle. For conditions with unknown proteins excluded, those proteins that were consistently classified as unknown were removed from the analysis. ER, endoplasmic reticulum; HPA, Human Protein Atlas; SVM, support vector machine; TAGM-MAP, t-augmented Gaussian mixture modeling with *maximum a posteriori* estimates.

To gauge the quality of our classifications, we compared those proteins that were consistently classified, that is, four of six replicates, for WT uninduced to several published spatial proteomes: MitoCarta2.0 database (23), a study of the mitochondrial matrix proteome (24), and a review of lysosome proteomic studies (25) (Fig. 5*A*). Examining those proteins from each study that were detected in our datasets, we found that TAGM-MAP consistently outperformed SVM for mitochondria but performed less well for lysosomes. We also compared only those proteins that received an organellar classification, that is, we excluded consensus unknown designations, to see if a focus on only proteins that remained classified would change the performance of SVM (*orange bars*) or TAGM-MAP (*dark orange bars*). SVM was more responsive to the exclusion of unknown proteins compared with TAGM-MAP, which is likely because of the lower proportion of unknown proteins in the TAGM-MAP uninduced condition.

We did a similar analysis for additional organelles by comparing to the HPA (26). To obtain a baseline to our analysis, we focused on those proteins considered by the HPA to be localized to a single organelle with high confidence (enhanced rating). Of those proteins, we then plotted the percentage that was similarly classified by SVM or TAGM-MAP (Fig. 5*B*). Again, we found that TAGM-MAP outperformed SVM for mitochondrial proteins, and we saw a similar trend for ER proteins, albeit to a lesser degree. Conversely, SVM outperformed TAGM-MAP in the Golgi apparatus, nucleus, peroxisomes, and plasma membrane, although only two proteins were considered for the peroxisome. Similar to our aforementioned observations, the exclusion of unknown proteins yielded a larger increase in percentage agreement for SVM (*orange bars versus blue bars*) than TAGM-MAP (*dark orange bars versus green bars*); this exclusion also increased the performance in the cytosol for SVM over TAGM-MAP. These data correspond well to those of Figure 4*A* where 114 proteins designated as unknown by SVM were classified as mitochondrial by TAGM-MAP. Similar trends were found within ΔNef data (supplemental Fig. S8). Taken together, this suggests that at least in this cell system and using these fractionation methods, TAGM-MAP is better suited for spatial proteomic studies focused on the mitochondria and ER, whereas SVM is better suited for studies of the Golgi, nucleus, and plasma membrane. This finding was surprising as we observed higher average QSep scores for the mitochondria and ER in WT replicates using SVM as compared with TAGM-MAP (supplemental Fig. S9), with less of a difference in ΔNef replicates (supplemental Fig. S10), which suggests an imperfect correlation between QSep scores and general accuracy for certain organelles.

### BANDLE Analysis of Row-Normalized WT Replicates Yielded the Best Agreement of HIV-Dependent Translocations With Known HIV Interactomes; Partial Overlap With ΔNEF Translocation Events

**Fig. 6:**
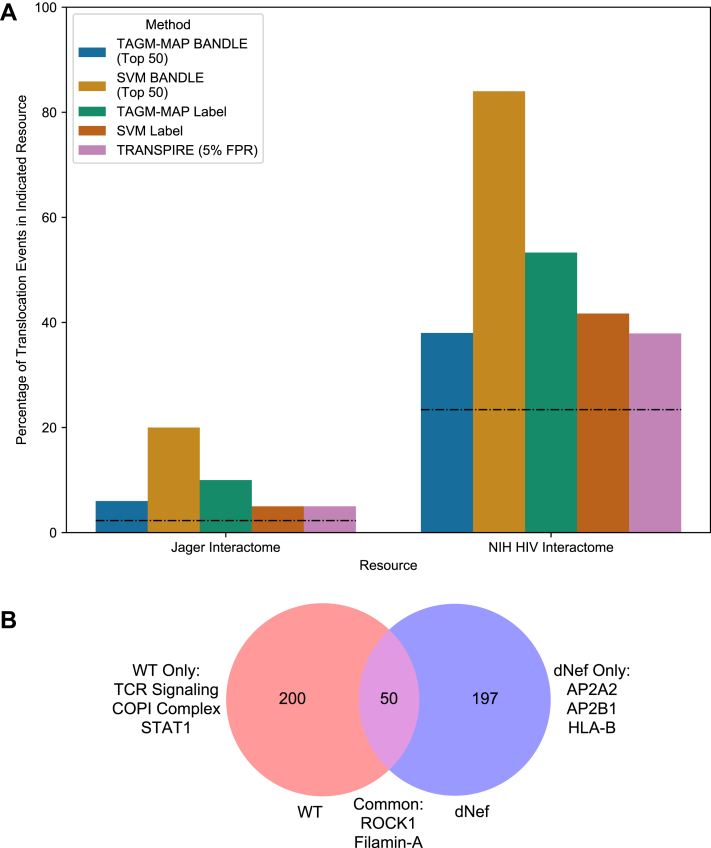
**Detection of protein translocations by SVM-based BANDLE analysis shows the highest rate of identifying known HIV interactors.***A*, the percentage of translocation events from each method that are in the Jӓger HIV interactome (27) (*left bars*) or the NIH HIV interactome (28) (*right bars*) is shown. *Dashed lines* indicate the percentage of events that would be expected by chance based on the proportion of the human proteome represented in each interactome. *B*, Venn diagram of top 250 events from SVM-based BANDLE for WT and ΔNef replicates. Three of the events from the ΔNef analysis were not detected by MS in WT replicates and were thus removed from consideration. BANDLE, Bayesian analysis of differential localization experiments; NIH, National Institutes of Health; SVM, support vector machine.

Following our analysis of classifiers, we examined various pipelines for identifying protein translocations: (1) we inputted our row-normalized (used for SVM) or PCA-normalized (used for TAGM-MAP) data into BANDLE (10), (2) we inputted SVM or TAGM-MAP classified data into a basic label-based analysis (7), and (3) we inputted unclassified data into TRANSPIRE (9) (Fig. 2*A*). We refer to the BANDLE analysis of row-normalized data as SVM BANDLE or SVM-based BANDLE and the BANDLE analysis of PCA-normalized data as TAGM-MAP BANDLE or TAGM-MAP-based BANDLE to emphasize the normalization technique used for each. For TRANSPIRE, we combined the organelles into five groups: (1) Golgi apparatus/plasma membrane/ER/peroxisomes/lysosomes, (2) cytosol/actin cytoskeleton/proteasome, (3) nucleus, (4) mitochondria, and (5) 40S/60S ribosome. This is in line with the authors' recommendation to combine similarly behaving organelles to increase translocation confidence (9), although in our case, we lose the ability to identify proteins moving between the membranous organelles most likely to be affected by Nef, that is, secretory organelles. To compare the performance of these five methods, we crossreferenced their translocation events against an HIV interactome derived from affinity purification-MS (AP-MS) (27) as well as the NIH HIV interactome (28). The AP-MS study is more stringent since it includes only those proteins that directly complex with HIV proteins, whereas the NIH HIV interactome includes proteins that are affected by HIV even in the absence of evidence for a direct interaction. We found that the percentage of events from each method that were in the interactomes was consistently above the threshold expected by chance (Fig. 6*A*, *dashed line*). Comparing the methods, the top 50 events from the SVM-based BANDLE analysis performed best for both interactomes with 20% and 84% of events in the study by Jӓger *et al.* (direct interactome by AP-MS) and NIH HIV interactome (functional as well as direct interactors), respectively. We conducted a similar translocation event analysis on our ΔNef-inducible line and found that SVM-based BANDLE was still the highest performer for the NIH HIV interactome but was only third best for the AP-MS interactome (supplemental Fig. S11).

The top 250 events from SVM-based BANDLE for WT and ΔNef were compared to see if the method could identify Nef-dependent translocations (Fig. 6*B*); events that were detected by MS in only WT or ΔNef replicates were removed to avoid detection bias. Of those events found only for WT, we observed several known Nef targets and cofactors: ZAP70 (38), Lck (18, 39), signal transducer and activator of transcription 1 (40), and coatomer complex I (coat protein complex I) (41, 42). Five separate proteins in the coat protein complex I appear together as well as three proteins from the T-cell signaling pathway, suggesting high coverage of perturbed complexes. For commonly shared translocation events, proteins involved in cytoskeletal organization were enriched. Disruption of the cytoskeleton following infection with HIV has been attributed to Nef among other viral proteins, but the enriched proteins here lacked known targets of Nef but instead included Rho-associated kinase 1, an interactor of HIV Tat, and filamin-A, an interactor of HIV Gag (43). We were surprised to see two components of the AP2 adaptor complex, known interactors of Nef (44), and HLA class B, a known target of Nef (45, 46), in the ΔNef-only translocations. The SVM classification for these select proteins and STRING diagrams of the full protein sets are shown in supplemental Figs. S12–S15. Notably, the SVM classifications rarely provided definitive organellar translocations for the events identified by BANDLE (supplemental Fig. S12). In some cases, this was due to the majority of replicates becoming unclassified in the induced condition. An interesting exception is filamin-A: although a translocation event in both WT and ΔNef cells occurs by BANDLE (Fig. 6*B*). By SVM classification, filamin-A moves from the actin cytoskeleton to the cytosol in cells expressing WT but not ΔNef (supplemental Fig. S12*K*). While the basis for such analytic discrepancies is unclear, taken together, these data suggest potential value in identifying novel HIV cofactors, targets, and interactors *via* BANDLE analysis of spatial proteomics data.

## Discussion

We have detailed here a comparison of computational methods within the field of spatial proteomics as an example and guide for researchers hoping to use these methods to better understand viral infection and replication. Extensive work in the field, particularly by the Lilley (2, 11, 22, 37, 47), Cristea (7, 8, 9), and Borner groups (4, 6, 48), along with their collaborators, offers a variety of established choices for fractionation, classification, and translocation identification methods. To build off of the work of the Cristea group with HCMV, we chose to examine HIV-1 as a model virus because of the existing wealth of knowledge on HIV-dependent protein interactions and translocations. We found in our T-cell line model and using differential centrifugation for cell fractionation that the optimal computational method for classification is organelle dependent: TAGM-MAP offered an advantage for mitochondrial and ER proteins, whereas SVM performed better for the Golgi apparatus, nucleus, and plasma membrane. For identifying translocations, BANDLE gave the highest agreement with known HIV biology (*i.e.*, published interactome data), particularly when coupled with row-normalized data.

The model of inducible HIV in Jurkat T cells used here has advantages and disadvantages. One advantage is that the system provides a highly homogenous population of HIV-expressing cells suitable for mass spectrometric analysis (13). A homogenous population is particularly important in spatial proteomic studies as mixed populations of cells might yield erroneous classifications of proteins because of mixtures of different states (47). Another advantage is scalability. These experiments required just over 3 × 10^8^ cells for each technical replicate or over 1 × 10^9^ cells for a single biological replicate, to be induced. In our initial attempts with fewer cells, centrifugation at higher relative centrifugal force (110,000*g* and 195,500*g*) yielded insufficient protein mass for quality control and MS (data not shown). This highlights an advantage of using this T-cell line compared with using primary CD4+ T cells (49), which in principle would be more relevant but would require at least 2 × 10^9^ cells and extraordinary viral inocula to achieve a high-multiplicity and synchronized infection. A disadvantage of using this T-cell system is that the cytoplasmic volume of the cell is relatively small. We required an order of magnitude in more cells for each technical replicate here than was used in the DOM studies of Itzhak *et al.*, who used HeLa cells with larger cytoplasm.

In addition to these technical considerations for modeling viral infection/expression, the choice of fractionation method has practical and computational implications. The use of differential centrifugation here and by Itzhak *et al.* (2) requires the downstream analysis of fewer fractions than gradient fractionation methods and is far less time intensive, resource intensive, and labor intensive. On the other hand, gradient fractionation methods seem to show increased resolution of protein classification (22). In an attempt to increase the organellar resolution of the DOM method, we used additional high-speed centrifugation steps to those described in the DOM method of Itzhak *et al.* and found a significant increase in overall organellar resolution using seven fractions as compared with the original five (Fig. 2). Previous work by the Lilley group comparing differential centrifugation and gradient-based methods for fractionation revealed comparable downstream results for the two methods using U-2 OS cells with differential centrifugation having a slight advantage in resolving the cytosol and proteasome (37), but whether this trend would hold in different cell types after viral infection or gene expression is unclear. Generalizable rules for spatial proteomics might require comparisons of various fractionation and computational methods in multiple systems, or perhaps more likely, the specific experimental system, and questions asked might be best addressed by a specific method. For example, to investigate translocations caused by HIV-1 Nef, better separation of membranous organelles (supplemental Figs. S5 and S6) might have yielded more Nef-specific translocations.

Our findings on classification consistency and accuracy might influence the choice of classifier, at least for this model system. We found that SVM yielded higher consistency in classification than TAGM-MAP, although both suffered similar losses in consistency following HIV expression. In cases where infection or viral expression is expected to introduce greater noise in the data, as seems to be the case here, SVM may be the better option as it yielded a higher starting point for consistency. If lower tolerance to noise is acceptable, TAGM-MAP offers an advantageous alternative for both the mitochondria and ER. TAGM-MAP also suffered less loss of protein classification to unknown designations for uninduced replicates, perhaps due in part to the threshold used here for retaining SVM classification. While we used a basic median SVM algorithm score threshold for each organelle (2) to allow for raw comparisons of classifiers to existing spatial proteomes, this might have been overly stringent for certain organelles, which would explain the higher number of unknown designated proteins for SVM. An alternative method would be to introduce an organelle-dependent threshold that would cap false positives by comparing classifier outputs to Gene Ontology analysis and published spatial proteomes; this method was employed previously by the Lilley group (1, 37). We further note the fact that while SVM showed generally higher QSep scores for the mitochondria and ER, it still underperformed compared with TAGM-MAP for these organelles. This suggested to us that organellar resolution as measured by QSep might be an imperfect measure of classification accuracy for a given organelle, a hypothesis that will need further examination.

Finally, the choice of translocation identification method requires consideration of several factors, the first of which is the experimental design. Part of BANDLE's power comes from its ability to factor multiple replicates of a condition into translocation event determination. Indeed, we saw a generally higher predictive power for BANDLE compared with other methods. The ranked list of output is also useful in cases where resources are limited and only a few proteins can be pursued. TRANSPIRE seemed to have poorer performance compared with other methods, but this might reflect our need to combine similarly fractionated organelle groups to reduce computational demand and increase resolution. In cases where individual organellar resolution is greater, TRANSPIRE might yield higher quality translocation events. Notably, both BANDLE and TRANSPIRE require intensive computational resources, with TRANSPIRE requiring supercomputer access for larger more complex datasets; the analyses performed here were done using two to three nodes with 200 Gb of RAM each and could be completed in the course of a day. In cases where computational power is limited, label-based methods would be suitable. Indeed, this method was employed by the Cristea group for their HCMV study with success (7).

A challenge not addressed here is how to handle changes in whole-organellar behavior within spatial proteomics, such as might be induced by viruses. Indeed, we observed such a change within our data: peroxisomal marker proteins shifted in their fractionation behavior (peak abundance occurring at a higher *g*-force) when WT HIV was induced, becoming very similar in their behavior to marker proteins of the ER (supplemental Fig. S5). This effect was not observed for ΔNef (supplemental Fig. S6). A previous discussion of this issue by the Lilley group (10) highlighted the various possible causes of whole-organellar changes—for example, differences in organelle protein content, lipid composition, or morphology—and their effect on the movement-reproducibility method of translocation identification, but these effects will vary depending on the choice of translocation identification method (5).

With these considerations in mind, our findings underscore that studies of spatial proteomics require careful consideration of the question at hand to inform the choice of methodology. Our work and that of others highlights the potential differences in organellar resolution that can result from the choice of fractionation and analytical methods. Interest in a particular organelle and in specific types of translocations will factor into the choice of methods. Our findings offer a reference point for studies of viral infection by spatial proteomics, for general studies of the spatial proteome, and for the study of additional gene dropout mutants of HIV-1.

## Data Availability

MS data (.RAW files and peptide identification tables) can be found on the ProteomeXchange database using project accession number PXD029956.

## Supplemental data

This article contains supplemental data (23, 24, 25, 26, 27, 28).

## Conflict of interest

The Krogan Laboratory has received research support from Vir Biotechnology and 10.13039/100007013F. Hoffmann-La Roche. N. J. K. has consulting agreements with the Icahn School of Medicine at Mount Sinai, New York, Maze Therapeutics, and Interline Therapeutics. He is a shareholder in Tenaya Therapeutics, Maze Therapeutics, and Interline Therapeutics, and a financially compensated Scientific Advisory Board Member for GEn1E Lifesciences, Inc. All other authors declare no competing interests.

## Supplemental Data

Supplemental Figures S1–S15

dNef1_IndA

dNef1_IndB

dNef1_IndC

dNef1_UnA

dNef1_UnB

dNef1_UnC

dNef2_IndA

dNef2_IndB

dNef2_IndC

dNef2_UnA

dNef2_UnB

dNef2_UnC

WT1_IndA

WT1_IndB

WT1_IndC

WT1_UnA

WT1_UnB

WT1_UnC

WT2_IndA

WT2_IndB

WT2_IndC

WT2_UnA

WT2_UnB

WT2_UnC
