# Supplementary material for: Comparative Analysis of T-Cell Spatial Proteomics and the Influence of HIV Expression
Source: Mol Cell Proteomics. 2022 Jan 8;21(3):100194. doi: 10.1016/j.mcpro.2022.100194 (PMC8956815; doi:10.1016/j.mcpro.2022.100194)
Supplement: Supplemental Figures S1–S15 [file mmc1.pdf]

Comparative Analysis of T-Cell Spatial Proteomics and the Influence of HIV Expression

Aaron L. Oom, Charlotte A. Stoneham, Mary K. Lewinski, Alicia Richards, Jacob M. Wozniak, Km Shams-Ud-Doha, David J. Gonzalez, Nevan J. Krogan, John Guatelli

Includes Supplemental Figures 1-15 (Protein ID Tables are in a separate .zip folder)

Figure S1

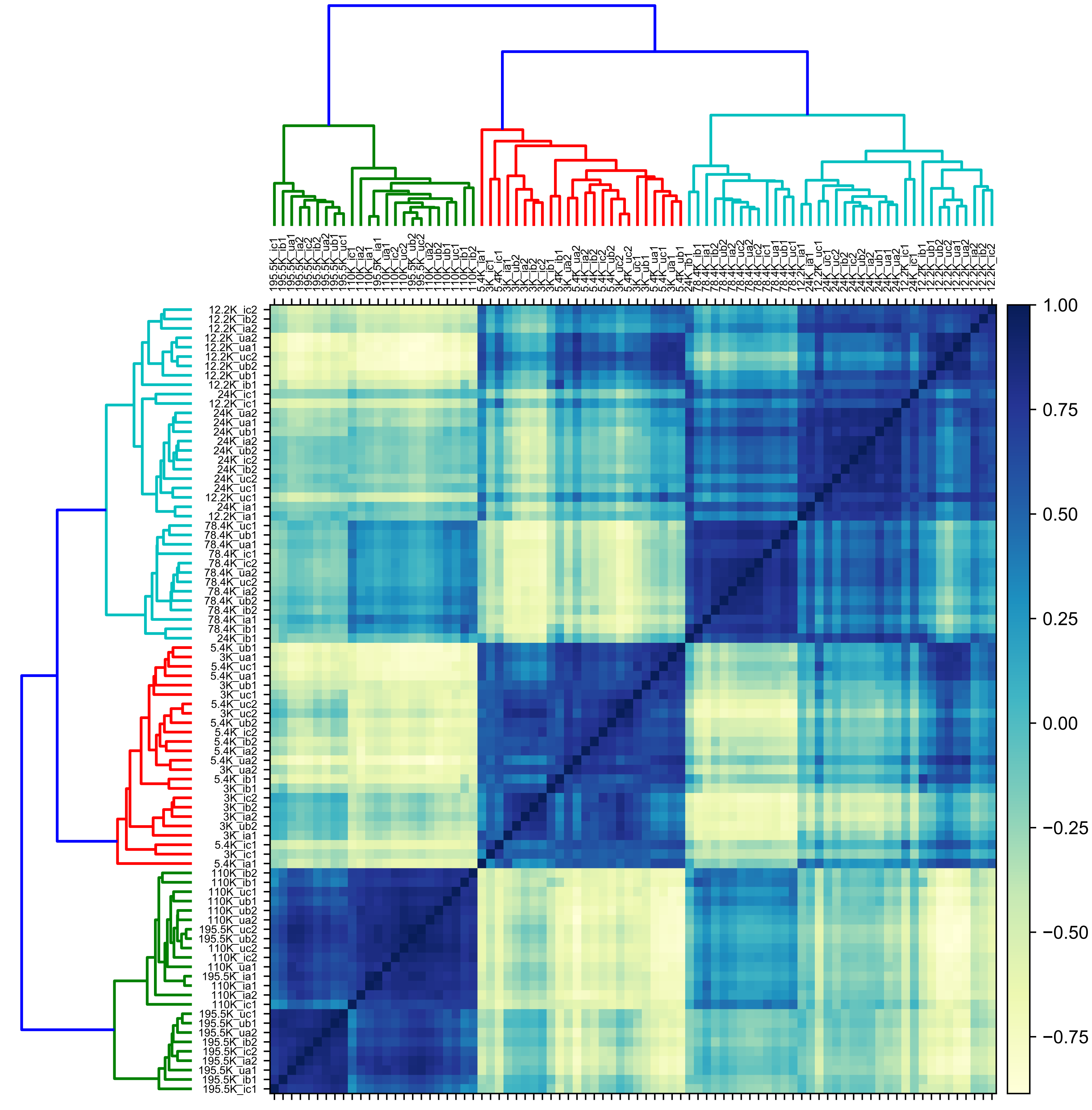
12.2K_ic2
12.2K_ib2
12.2K_ia2
12.2K_ua2
12.2K_ua1
12.2K_uc2
12.2K_ub2
12.2K_ub1
12.2K_ib1
24K_ic1
12.2K_ic1
24K_ua2
24K_ua1
24K_ub1
24K_ia2
24K_ub2
24K_ic2
24K_ib2
24K_uc2
24K_uc1
12.2K_uc1
24K_ia1
12.2K_ia1
78.4K_uc1
78.4K_ub1
78.4K_ua1
78.4K_ic1
78.4K_ic2
78.4K_ua2
78.4K_uc2
78.4K_ia2
78.4K_ub2
78.4K_ib2
78.4K_ia1
78.4K_ib1
24K_ib1
5.4K_ub1
3K_ua1
5.4K_uc1
5.4K_ua1
3K_ub1
3K_uc1
5.4K_uc2
3K_uc2
5.4K_ub2
5.4K_ic2
5.4K_ib2
5.4K_ia2
5.4K_ua2
3K_ua2
5.4K_ib1
3K_ib1
3K_ic2
3K_ib2
3K_ia2
3K_ub2
3K_ia1
5.4K_ic1
3K_ic1
5.4K_ia1
110K_ib2
110K_ib1
110K_uc1
110K_ub1
110K_ub2
110K_ua2
195.5K_uc2
195.5K_ub2
110K_uc2
110K_ic2
110K_ua1
195.5K_ia1
110K_ia1
110K_ia2
110K_ic1
195.5K_uc1
195.5K_ub1
195.5K_ua2
195.5K_ib2
195.5K_ic2
195.5K_ia2
195.5K_ua1
195.5K_ib1
195.5K_ic1
1.00
0.75
0.50
0.25
0.00
−0.25
−0.50
−0.75

Supplemental Figure S1: Correlation analysis for WT cell fractions. Spearman correlation between individual fractions of both WT biological replicates. Fractions are labeled with a three-character code after the *g*-force: “u” or “i” for uninduced or induced, respectively; “a”, “b”, or “c” for the technical replicate; and “1” or “2” for the biological replicate. Only proteins detected in all WT replicates are considered here (4,765 proteins).

Figure S2

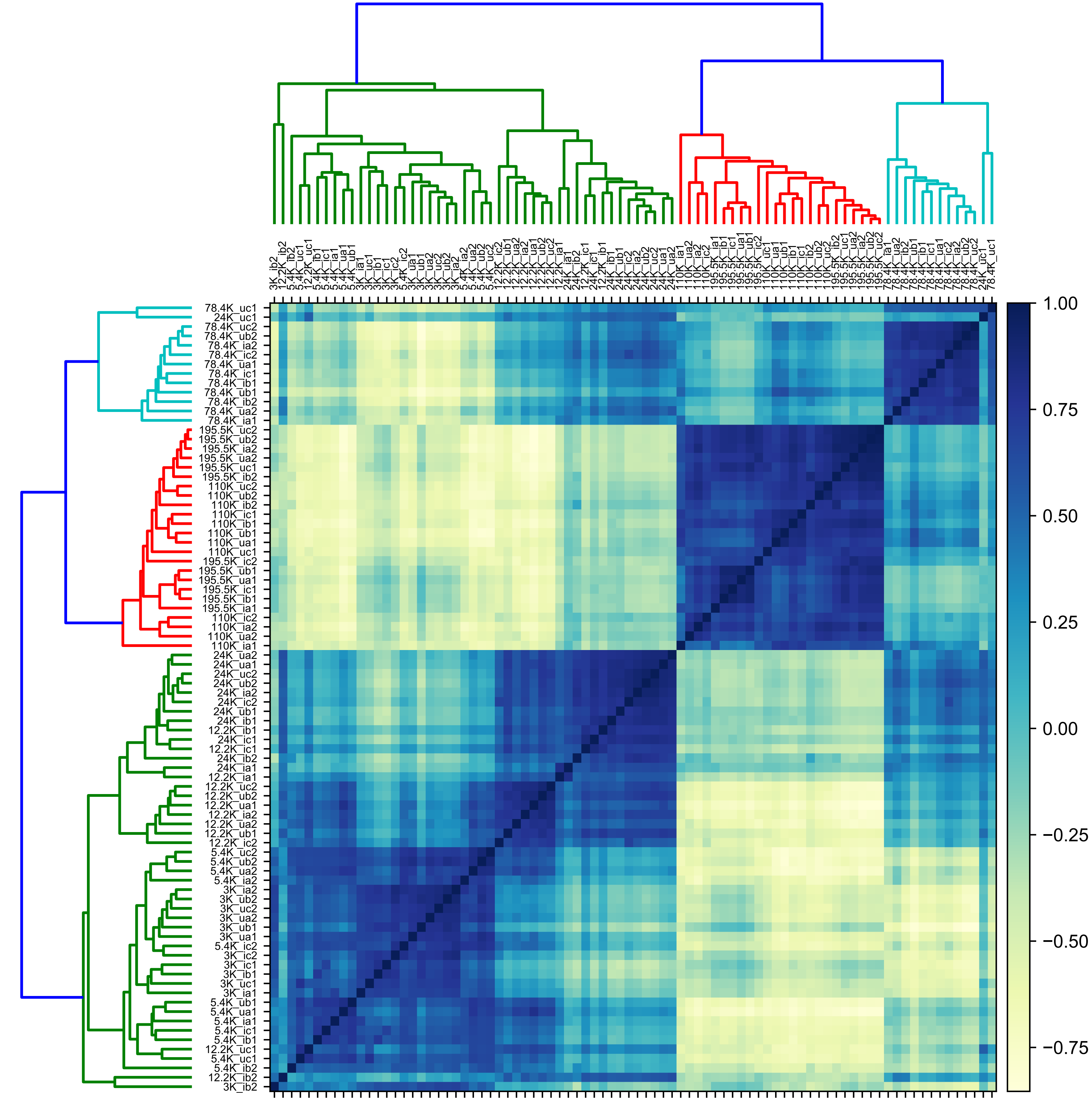
78.4K_uc1
24K_uc1
78.4K_uc2
78.4K_ub2
78.4K_ia2
78.4K_ic2
78.4K_ua1
78.4K_ic1
78.4K_ib1
78.4K_ub1
78.4K_ib2
78.4K_ua2
78.4K_ia1
195.5K_uc2
195.5K_ub2
195.5K_ia2
195.5K_ua2
195.5K_uc1
195.5K_ib2
110K_uc2
110K_ub2
110K_ib2
110K_ic1
110K_ib1
110K_ub1
110K_ua1
110K_uc1
195.5K_ic2
195.5K_ub1
195.5K_ua1
195.5K_ic1
195.5K_ib1
195.5K_ia1
110K_ic2
110K_ia2
110K_ua2
110K_ia1
24K_ua2
24K_ua1
24K_uc2
24K_ub2
24K_ia2
24K_ic2
24K_ub1
24K_ib1
12.2K_ib1
24K_ic1
12.2K_ic1
24K_ib2
24K_ia1
12.2K_ia1
12.2K_uc2
12.2K_ub2
12.2K_ua1
12.2K_ia2
12.2K_ua2
12.2K_ub1
12.2K_ic2
5.4K_uc2
5.4K_ub2
5.4K_ua2
5.4K_ia2
3K_ia2
3K_ub2
3K_uc2
3K_ua2
3K_ub1
3K_ua1
5.4K_ic2
3K_ic2
3K_ic1
3K_ib1
3K_uc1
3K_ia1
5.4K_ub1
5.4K_ua1
5.4K_ia1
5.4K_ic1
5.4K_ib1
12.2K_uc1
5.4K_uc1
5.4K_ib2
12.2K_ib2
3K_ib2
1.00
0.75
0.50
0.25
0.00
−0.25
−0.50
−0.75

Supplemental Figure S2: Correlation analysis for ΔNef cell fractions. Spearman correlation between individual fractions of both ΔNef biological replicates. Fractions are labeled with a three-character code after the *g*-force: “u” or “i” for uninduced or induced, respectively; “a”, “b”, or “c” for the technical replicate; and “1” or “2” for the biological replicate. Only proteins detected in all ΔNef replicates are considered here (4,173 proteins).

Figure S3

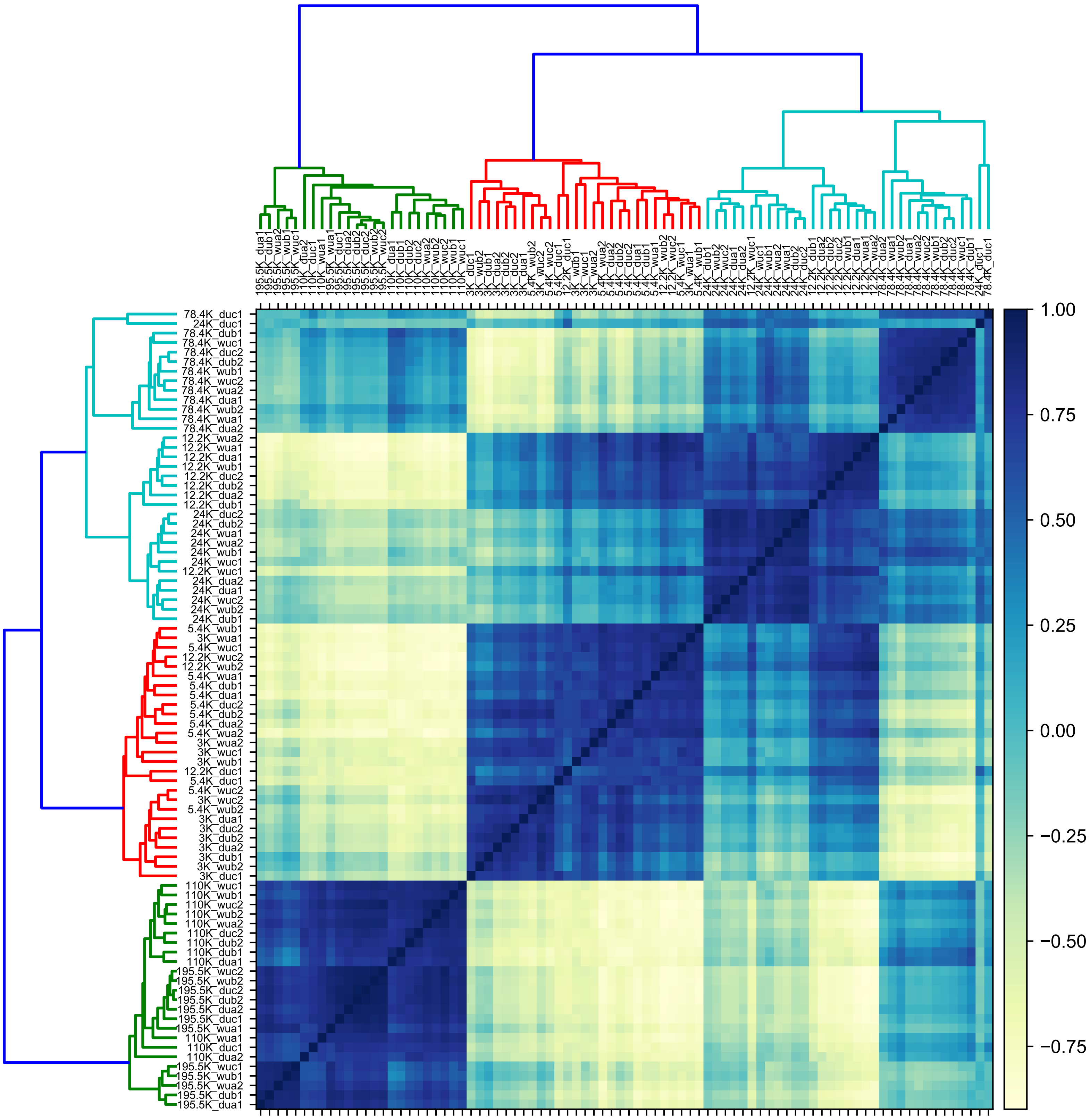
78.4K_duc1
24K_duc1
78.4K_dub1
78.4K_wuc1
78.4K_duc2
78.4K_dub2
78.4K_wub1
78.4K_wuc2
78.4K_wua2
78.4K_dua1
78.4K_wub2
78.4K_wua1
78.4K_dua2
12.2K_wua2
12.2K_wua1
12.2K_dua1
12.2K_wub1
12.2K_duc2
12.2K_dub2
12.2K_dua2
12.2K_dub1
24K_duc2
24K_dub2
24K_wua1
24K_wua2
24K_wub1
24K_wuc1
12.2K_wuc1
24K_dua2
24K_dua1
24K_wuc2
24K_wub2
24K_dub1
5.4K_wub1
3K_wua1
5.4K_wuc1
12.2K_wuc2
12.2K_wub2
5.4K_wua1
5.4K_dub1
5.4K_dua1
5.4K_duc2
5.4K_dub2
5.4K_dua2
5.4K_wua2
3K_wua2
3K_wuc1
3K_wub1
12.2K_duc1
5.4K_duc1
5.4K_wuc2
3K_wuc2
5.4K_wub2
3K_dua1
3K_duc2
3K_dub2
3K_dua2
3K_dub1
3K_wub2
3K_duc1
110K_wuc1
110K_wub1
110K_wuc2
110K_wub2
110K_wua2
110K_duc2
110K_dub2
110K_dub1
110K_dua1
195.5K_wuc2
195.5K_wub2
195.5K_duc2
195.5K_dub2
195.5K_dua2
195.5K_duc1
195.5K_wua1
110K_wua1
110K_duc1
110K_dua2
195.5K_wuc1
195.5K_wub1
195.5K_wua2
195.5K_dub1
195.5K_dua1
1.00
0.75
0.50
0.25
0.00
−0.25
−0.50
−0.75

Supplemental Figure S3: Correlation analysis for WT and ΔNef cell fractions. Spearman correlation between all individual fractions of uninduced condition. Fractions are labeled with a four-character code after the *g*-force: “w” or “d” for WT or ΔNef, “u” or “i” for uninduced or induced, respectively; “a”, “b”, or “c” for the technical replicate; and “1” or “2” for the biological replicate. Only proteins detected in all WT and ΔNef replicates are considered here (3,739 proteins).

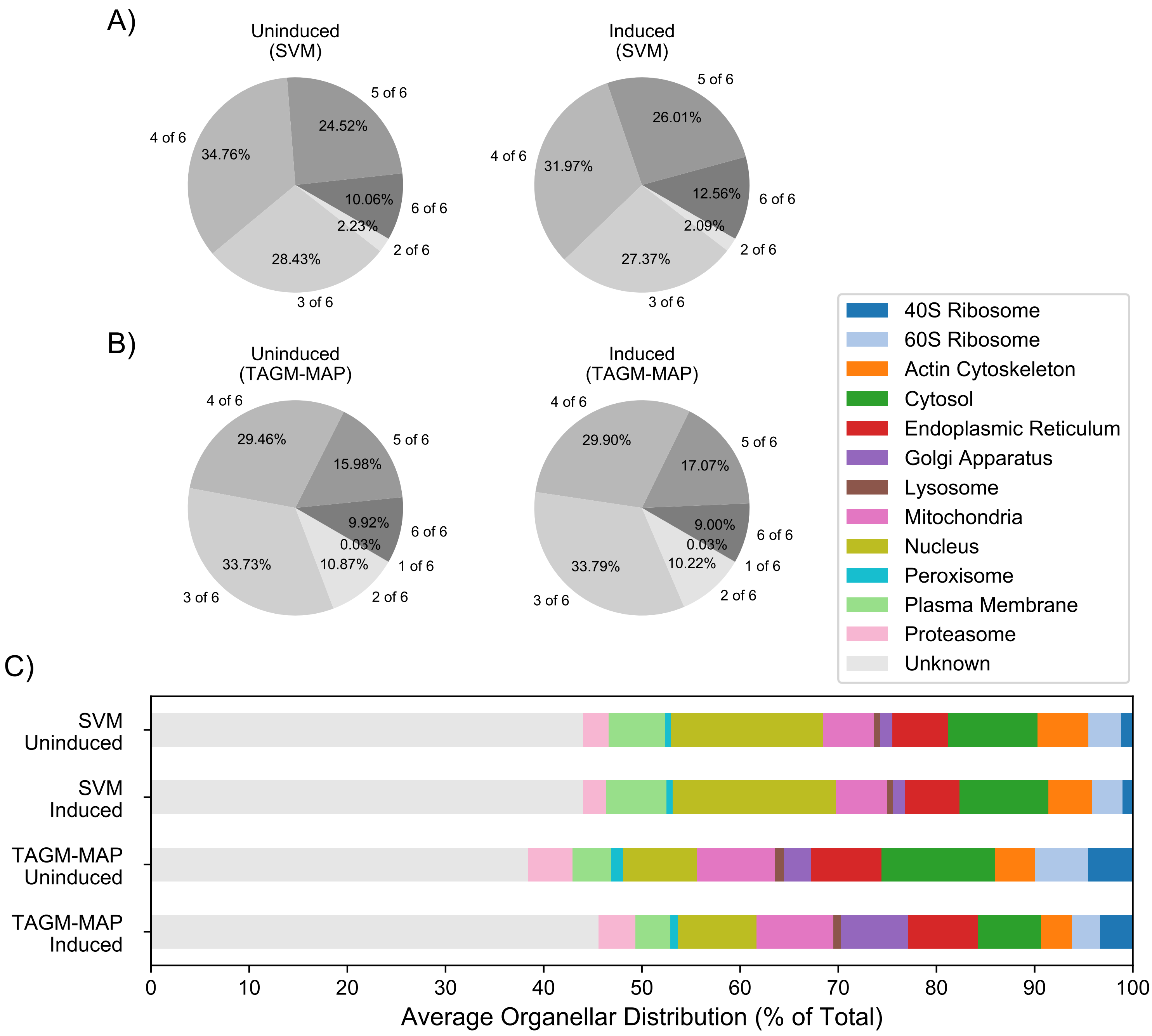
A)
Uninduced
(SVM)
4 of 6
34.76%
5 of 6
24.52%
10.06%
6 of 6
2.23%
2 of 6
28.43%
3 of 6
Induced
(SVM)
5 of 6
26.01%
4 of 6
31.97%
12.56%
6 of 6
2.09%
2 of 6
27.37%
3 of 6
B)
Uninduced
(TAGM-MAP)
4 of 6
29.46%
5 of 6
15.98%
9.92%
6 of 6
0.03%
1 of 6
33.73%
10.87%
3 of 6
2 of 6
Induced
(TAGM-MAP)
4 of 6
29.90%
5 of 6
17.07%
9.00%
6 of 6
0.03%
1 of 6
33.79%
10.22%
3 of 6
2 of 6
40S Ribosome
60S Ribosome
Actin Cytoskeleton
Cytosol
Endoplasmic Reticulum
Golgi Apparatus
Lysosome
Mitochondria
Nucleus
Peroxisome
Plasma Membrane
Proteasome
Unknown
C)
SVM
Uninduced
SVM
Induced
TAGM-MAP
Uninduced
TAGM-MAP
Induced
0
10
20
30
40
50
60
70
80
90
100
Average Organellar Distribution (% of Total)

<u>Supplemental Figure S4:</u> Classification with SVM shows greater consistency than TAGM-MAP classification for ΔNef replicates. A) Proteins were classified by SVM and the most frequent organellar classification was identified along with its frequency, i.e. number of technical replicates classified as such. Left pie chart shows consistency of classification for WT uninduced replicates and right pie chart shows WT induced replicates. B) Same as A), but classification by TAGM-MAP. C) Average distribution of proteins across organelles for each indicated condition. All charts consider the same common proteins found across all ΔNef replicates (4,173 proteins).

Figure S5

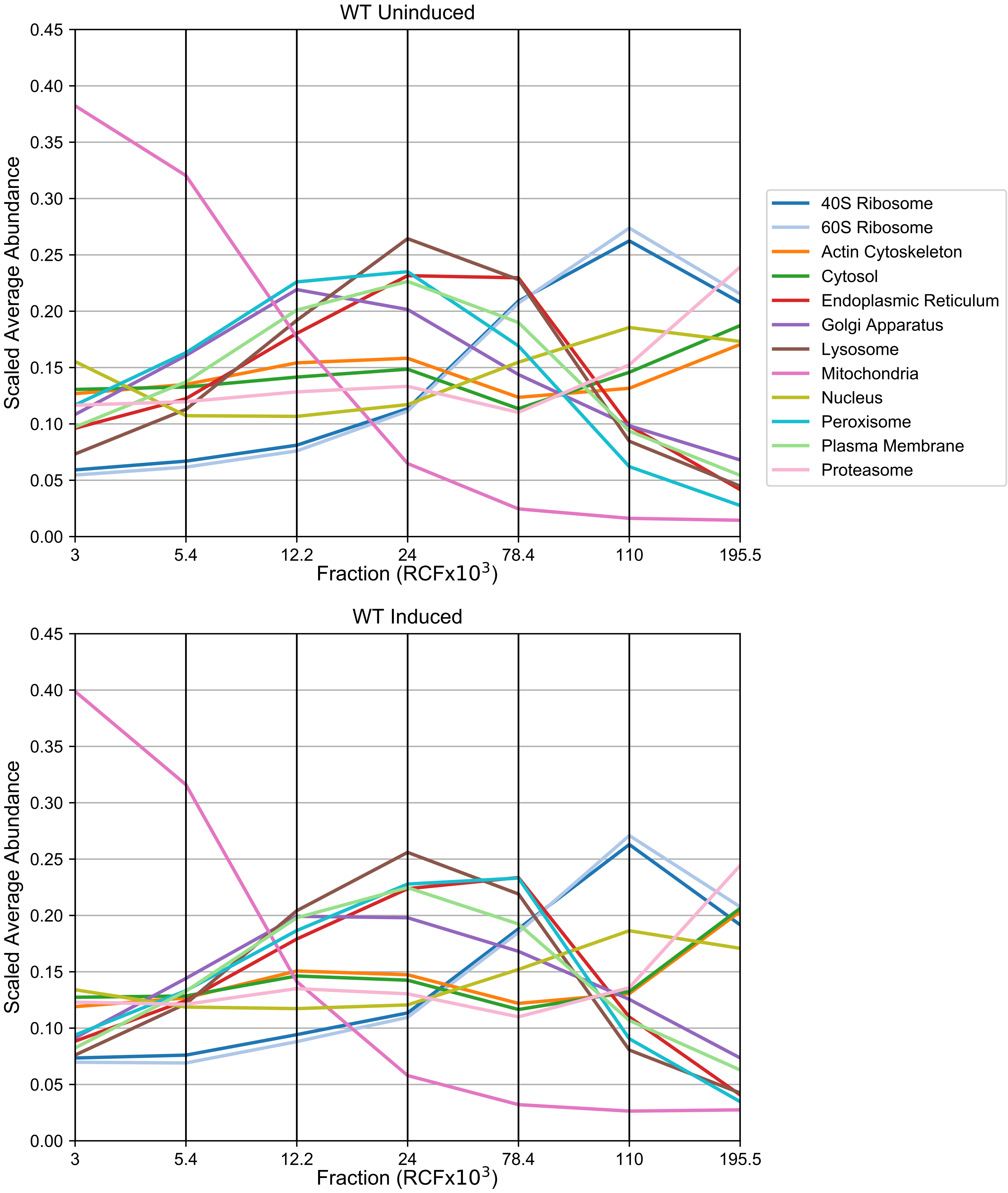
WT Uninduced
0.45
0.40
0.35
0.30
0.25
0.20
0.15
0.10
0.05
0.00
Scaled Average Abundance
3
5.4
12.2
24
78.4
110
195.5
Fraction (RCFx10^3)
40S Ribosome
60S Ribosome
Actin Cytoskeleton
Cytosol
Endoplasmic Reticulum
Golgi Apparatus
Lysosome
Mitochondria
Nucleus
Peroxisome
Plasma Membrane
Proteasome
WT Induced
0.45
0.40
0.35
0.30
0.25
0.20
0.15
0.10
0.05
0.00
Scaled Average Abundance
3
5.4
12.2
24
78.4
110
195.5
Fraction (RCFx10^3)

Supplemental Figure S5: Marker profiles of WT replicates. Average behavior of markers for each organelle have been charted for uninduced (top) and induced (bottom) replicates. Values were row-normalized prior to averaging. The “hsap” marker set from pRoloc was used.

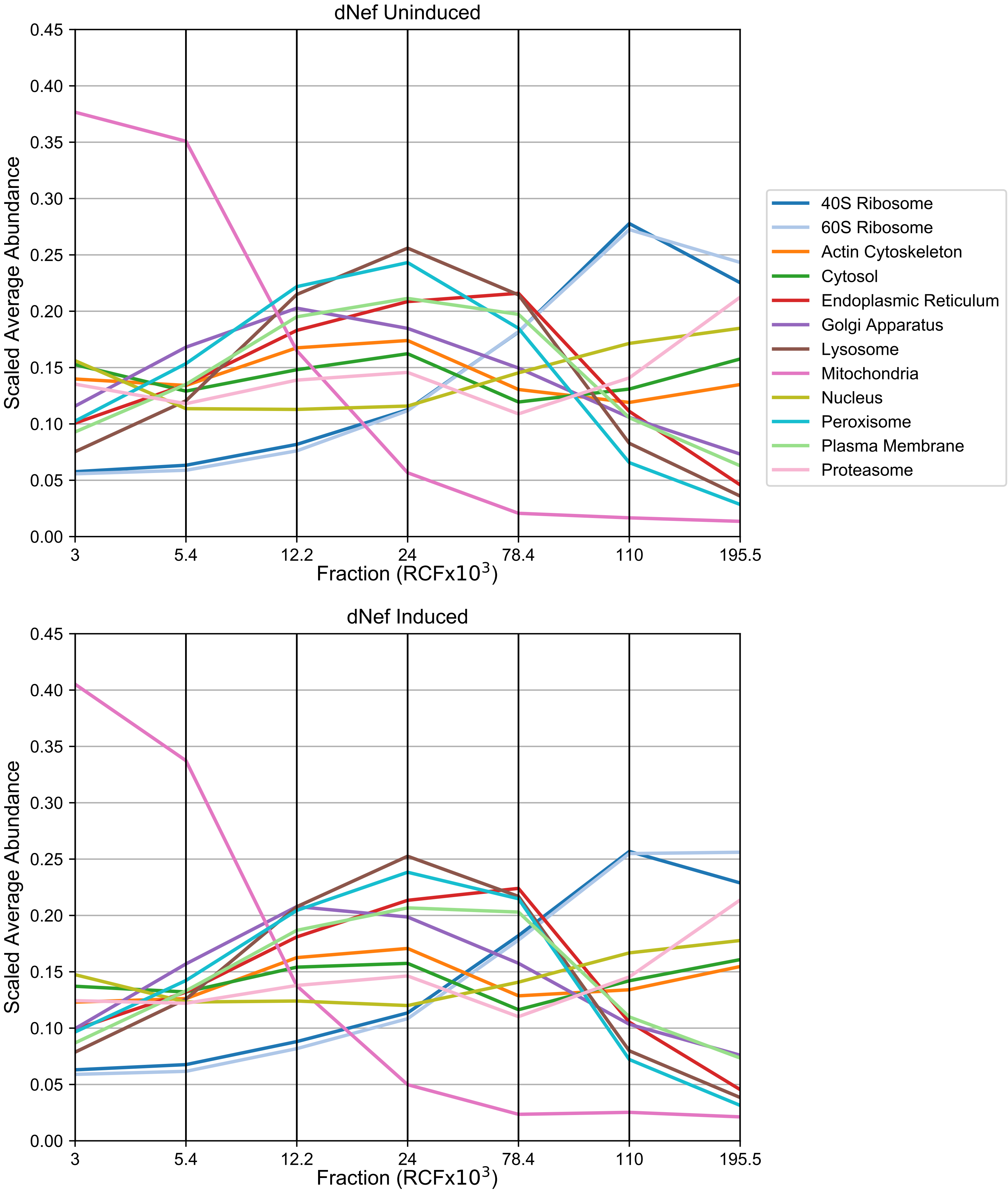
dNef Uninduced
Scaled Average Abundance
0.45
0.40
0.35
0.30
0.25
0.20
0.15
0.10
0.05
0.00
3
5.4
12.2
24
78.4
110
195.5
Fraction (RCFx10^3)
40S Ribosome
60S Ribosome
Actin Cytoskeleton
Cytosol
Endoplasmic Reticulum
Golgi Apparatus
Lysosome
Mitochondria
Nucleus
Peroxisome
Plasma Membrane
Proteasome
dNef Induced
Scaled Average Abundance
Fraction (RCFx10^3)

Supplemental Figure S6: Marker profiles of ΔNef replicates. Average behavior of markers for each organelle have been charted for uninduced (top) and induced (bottom) replicates. Values were row-normalized prior to averaging. The “hsap” marker set from pRoloc was used.

Figure S7

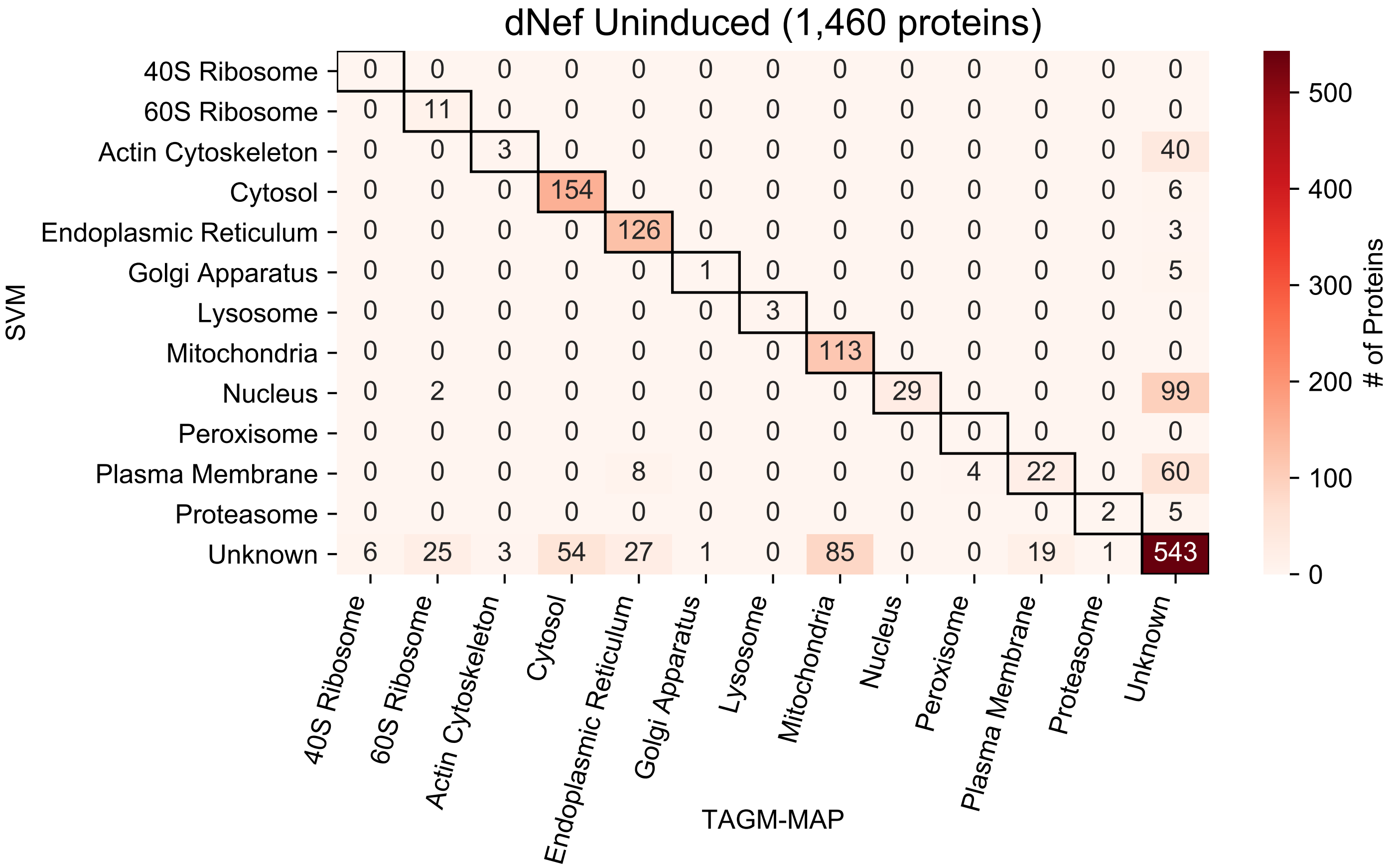
dNef Uninduced (1,460 proteins)
SVM
TAGM-MAP
# of Proteins
40S Ribosome 0 0 0 0 0 0 0 0 0 0 0 0 0
60S Ribosome 0 11 0 0 0 0 0 0 0 0 0 0 0
Actin Cytoskeleton 0 0 3 0 0 0 0 0 0 0 0 0 40
Cytosol 0 0 0 154 0 0 0 0 0 0 0 0 6
Endoplasmic Reticulum 0 0 0 0 126 0 0 0 0 0 0 0 3
Golgi Apparatus 0 0 0 0 0 1 0 0 0 0 0 0 5
Lysosome 0 0 0 0 0 0 3 0 0 0 0 0 0
Mitochondria 0 0 0 0 0 0 0 113 0 0 0 0 0
Nucleus 0 2 0 0 0 0 0 0 29 0 0 0 99
Peroxisome 0 0 0 0 0 0 0 0 0 0 0 0 0
Plasma Membrane 0 0 0 0 8 0 0 0 0 4 22 0 60
Proteasome 0 0 0 0 0 0 0 0 0 0 0 2 5
Unknown 6 25 3 54 27 1 0 85 0 0 19 1 543
40S Ribosome
60S Ribosome
Actin Cytoskeleton
Cytosol
Endoplasmic Reticulum
Golgi Apparatus
Lysosome
Mitochondria
Nucleus
Peroxisome
Plasma Membrane
Proteasome
Unknown
0
100
200
300
400
500

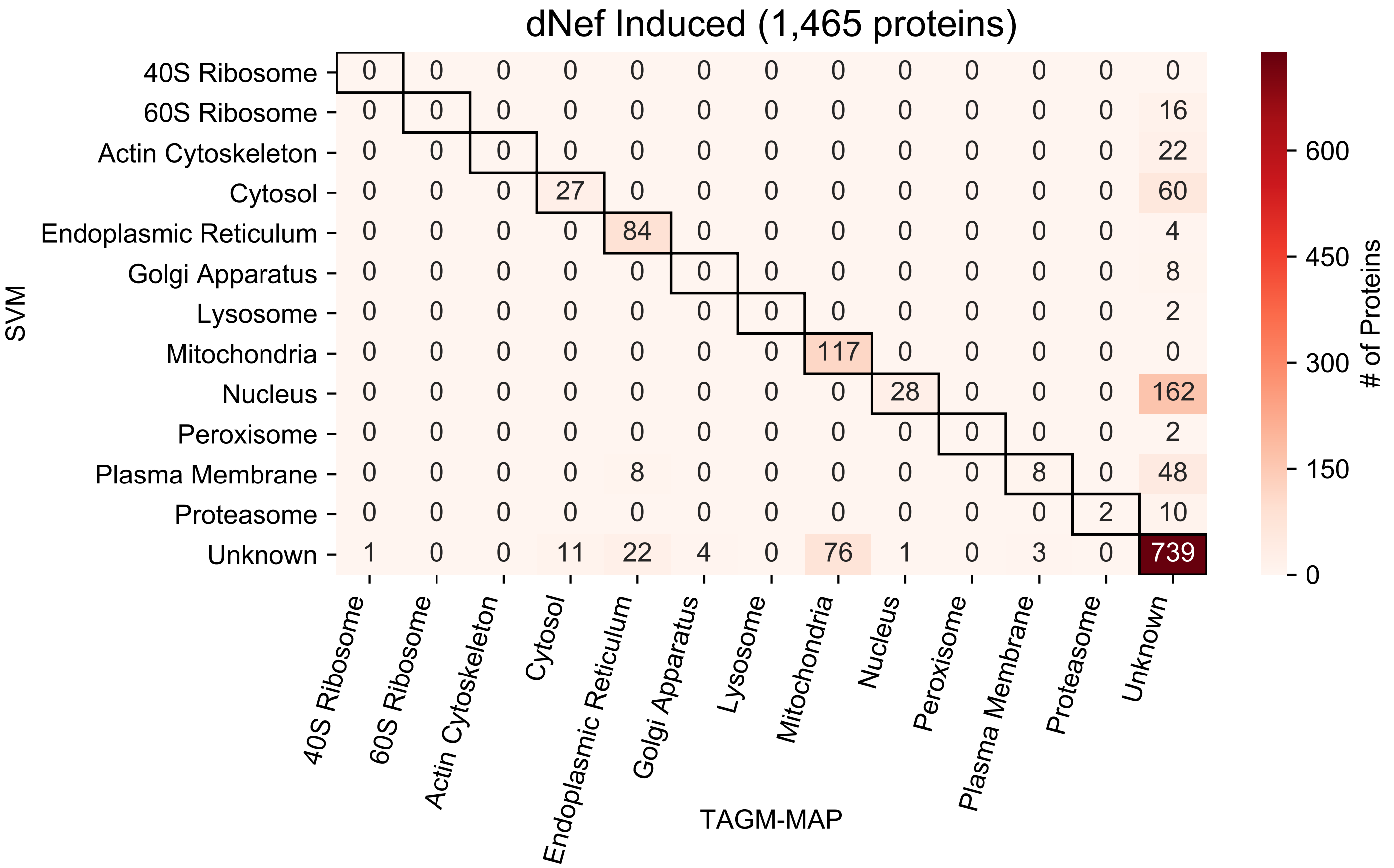
dNef Induced (1,465 proteins)
SVM
TAGM-MAP
# of Proteins
40S Ribosome 0 0 0 0 0 0 0 0 0 0 0 0 0
60S Ribosome 0 0 0 0 0 0 0 0 0 0 0 0 16
Actin Cytoskeleton 0 0 0 0 0 0 0 0 0 0 0 0 22
Cytosol 0 0 0 27 0 0 0 0 0 0 0 0 60
Endoplasmic Reticulum 0 0 0 0 84 0 0 0 0 0 0 0 4
Golgi Apparatus 0 0 0 0 0 0 0 0 0 0 0 0 8
Lysosome 0 0 0 0 0 0 0 0 0 0 0 0 2
Mitochondria 0 0 0 0 0 0 0 117 0 0 0 0 0
Nucleus 0 0 0 0 0 0 0 0 28 0 0 0 162
Peroxisome 0 0 0 0 0 0 0 0 0 0 0 0 2
Plasma Membrane 0 0 0 0 8 0 0 0 0 0 8 0 48
Proteasome 0 0 0 0 0 0 0 0 0 0 0 2 10
Unknown 1 0 0 11 22 4 0 76 1 0 3 0 739
40S Ribosome
60S Ribosome
Actin Cytoskeleton
Cytosol
Endoplasmic Reticulum
Golgi Apparatus
Lysosome
Mitochondria
Nucleus
Peroxisome
Plasma Membrane
Proteasome
Unknown
0
150
300
450
600

Supplemental Figure S7: Concordance of SVM and TAGM-MAP classifications for ΔNef replicates depend on organelle and expression of HIV. A) Heat map of common proteins that were consistently classified (proteins classified consistently in at least 4 of 6 replicates) by both SVM and TAGM-MAP for uninduced condition. Annotations indicate number of proteins in a given scenario. B) Same as A) for induced condition.

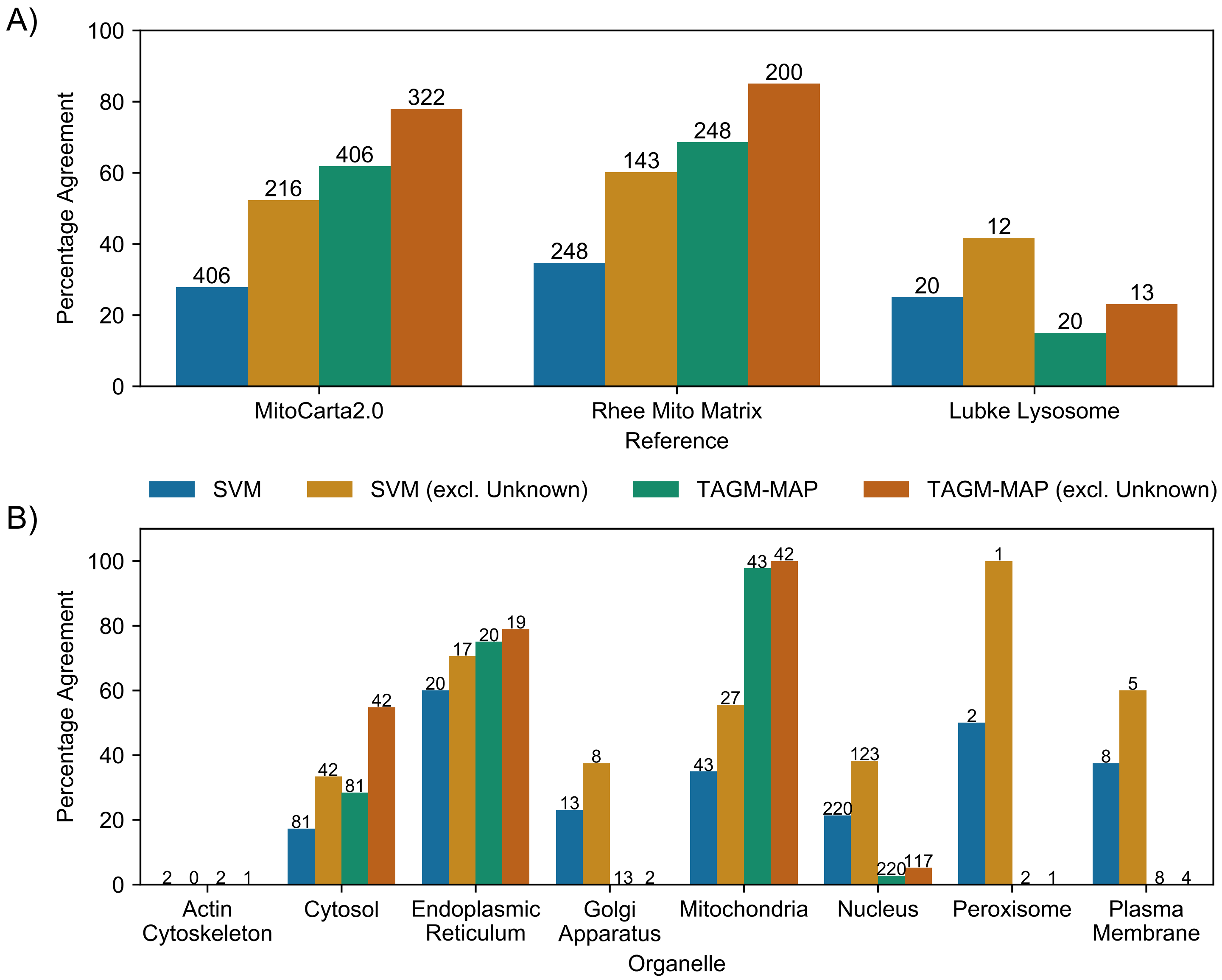
A)
Percentage Agreement
Reference
MitoCarta2.0
Rhee Mito Matrix
Lubke Lysosome
406
216
406
322
248
143
248
200
20
12
20
13
SVM
SVM (excl. Unknown)
TAGM-MAP
TAGM-MAP (excl. Unknown)
B)
Percentage Agreement
Organelle
Actin Cytoskeleton
Cytosol
Endoplasmic Reticulum
Golgi Apparatus
Mitochondria
Nucleus
Peroxisome
Plasma Membrane

Supplemental Figure S8: Validation of protein classification of ΔNef replicates reveals higher performance for ER and mitochondria using TAGM-MAP, but better performance for Golgi apparatus, nucleus, and plasma membrane using SVM. A) Percentage of detected proteins from MitoCarta2.0 database[23], Rhee et al. mitochondrial matrix study[24], or Lubke lysosome proteome[25] that were consistently classified (proteins classified consistently in at least 4 of 6 replicates) in line with the respective reference. Numbers above bars indicate the total number of proteins from that reference that were detected and classified for a given method. B) Proteins classified here were cross-referenced against the Human Protein Atlas[26] and any protein considered to be singularly localized with an Enhanced rating was kept. The percentage of these proteins that were consistently classified by SVM or TAGM-MAP into the HPA-designated organelle is shown. Numbers above bars indicate the number of HPA proteins considered for each organelle. For conditions with Unknown proteins excluded, those proteins that were consistently classified as Unknown were removed from the analysis.

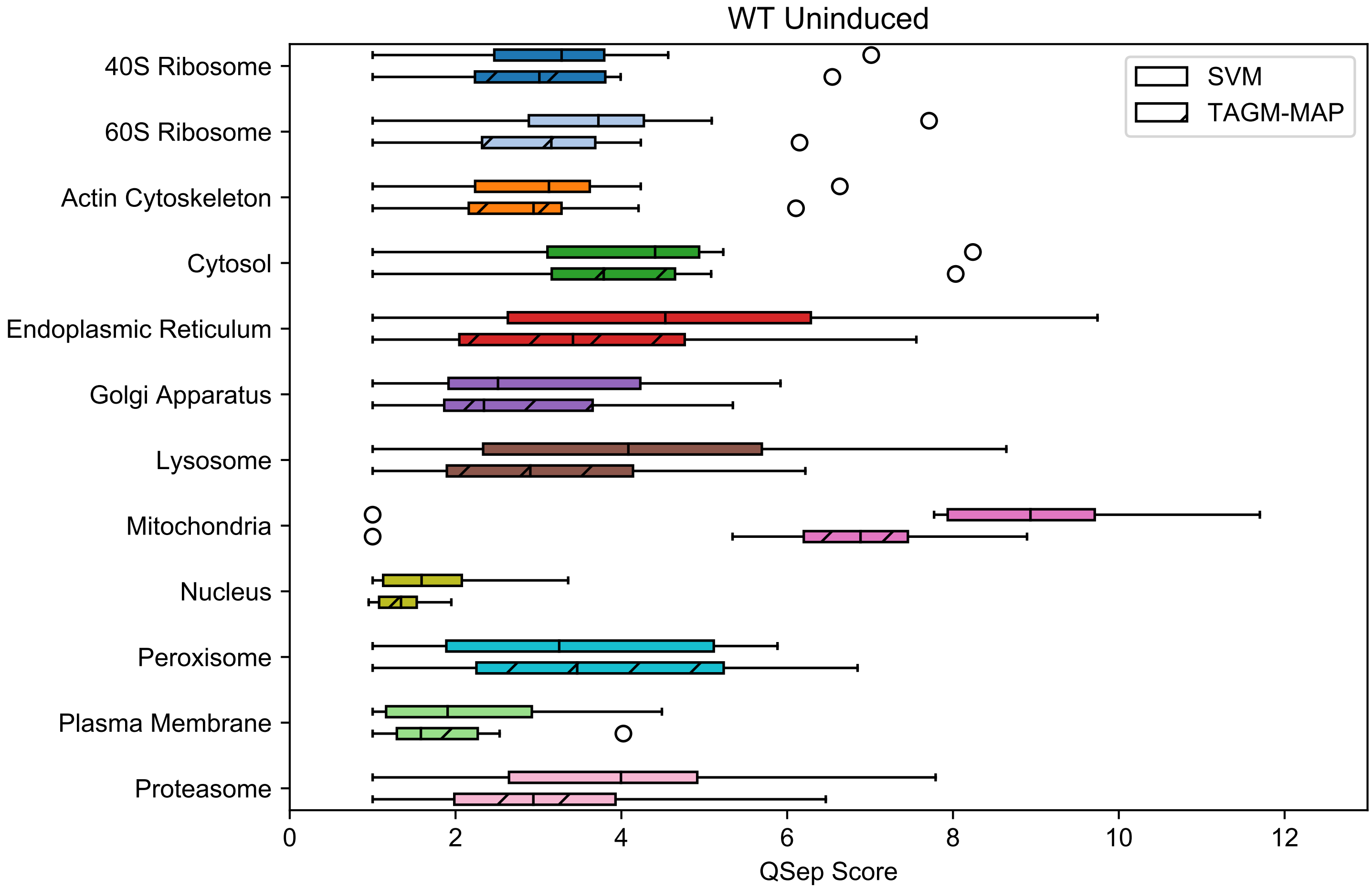
WT Uninduced
40S Ribosome
60S Ribosome
Actin Cytoskeleton
Cytosol
Endoplasmic Reticulum
Golgi Apparatus
Lysosome
Mitochondria
Nucleus
Peroxisome
Plasma Membrane
Proteasome
SVM
TAGM-MAP
0
2
4
6
8
10
12
QSep Score

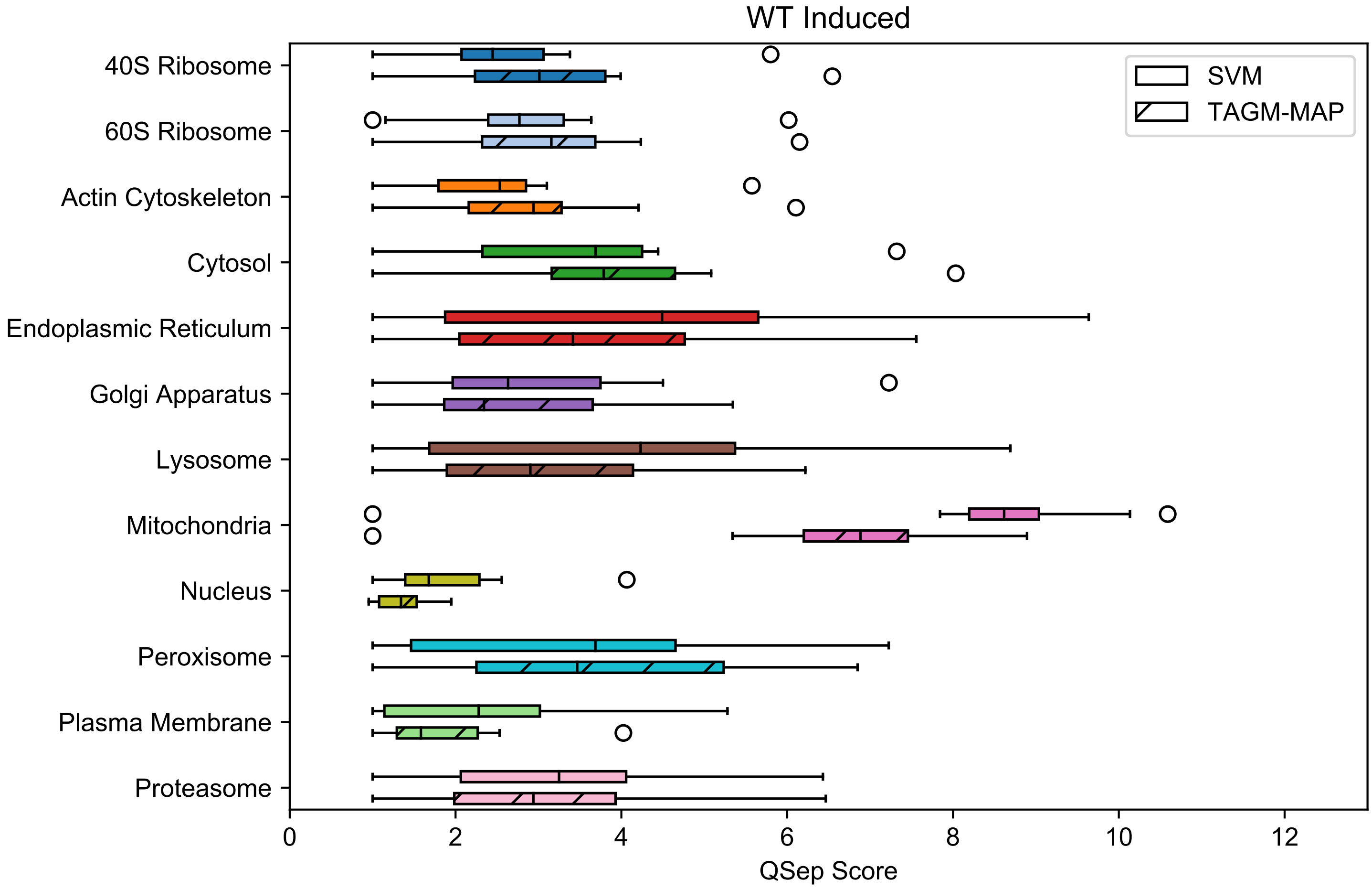
WT Induced
40S Ribosome
60S Ribosome
Actin Cytoskeleton
Cytosol
Endoplasmic Reticulum
Golgi Apparatus
Lysosome
Mitochondria
Nucleus
Peroxisome
Plasma Membrane
Proteasome
SVM
TAGM-MAP
0
2
4
6
8
10
12
QSep Score

Supplemental Figure S9: SVM classification shows generally higher organellar QSep scores for WT replicates. The individual organellar QSep scores, i.e. distance between organelle A and B, were averaged across uninduced replicates (top) or induced replicates (bottom) then plotted.

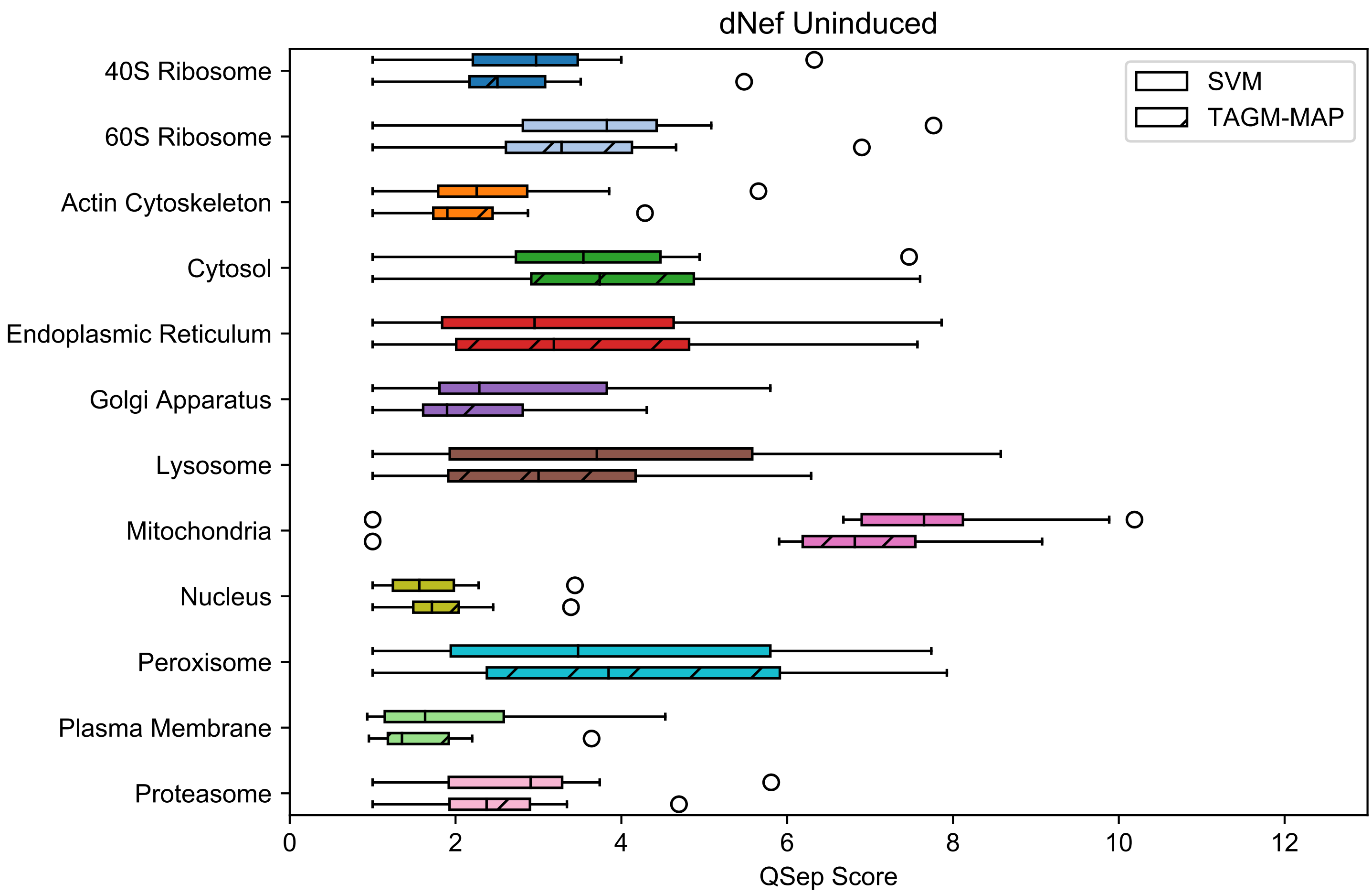
dNef Uninduced
40S Ribosome
60S Ribosome
Actin Cytoskeleton
Cytosol
Endoplasmic Reticulum
Golgi Apparatus
Lysosome
Mitochondria
Nucleus
Peroxisome
Plasma Membrane
Proteasome
SVM
TAGM-MAP
0
2
4
6
8
10
12
QSep Score

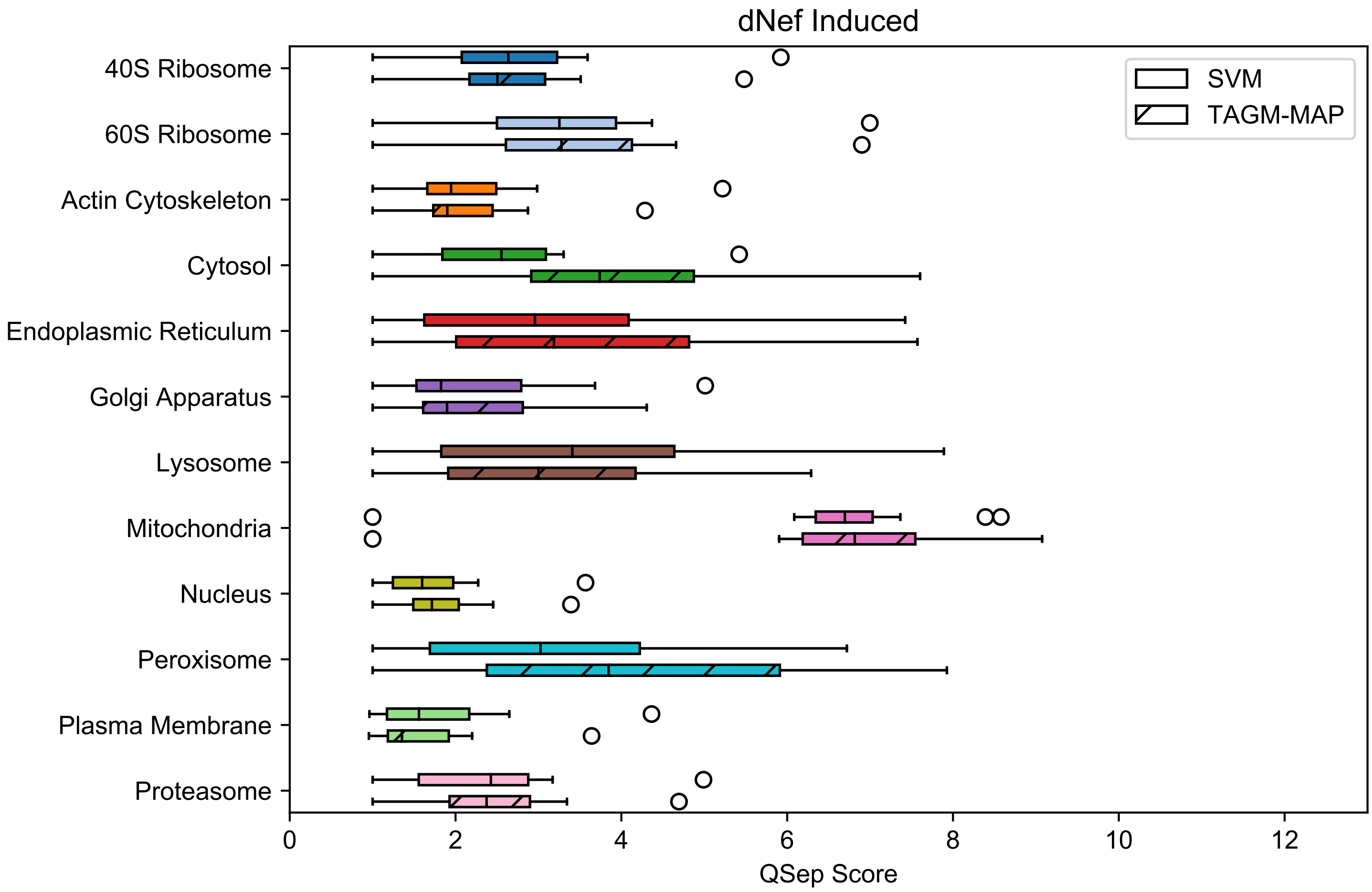
dNef Induced
40S Ribosome
60S Ribosome
Actin Cytoskeleton
Cytosol
Endoplasmic Reticulum
Golgi Apparatus
Lysosome
Mitochondria
Nucleus
Peroxisome
Plasma Membrane
Proteasome
SVM
TAGM-MAP
0
2
4
6
8
10
12
QSep Score

Supplemental Figure S10: SVM and TAGM-MAP classification show mixed results for organellar resolution by QSep scores for ΔNef replicates. The individual organellar QSep scores, i.e. distance between organelle A and B, were averaged across uninduced replicates (top) or induced replicates (bottom) then plotted.

Figure S11

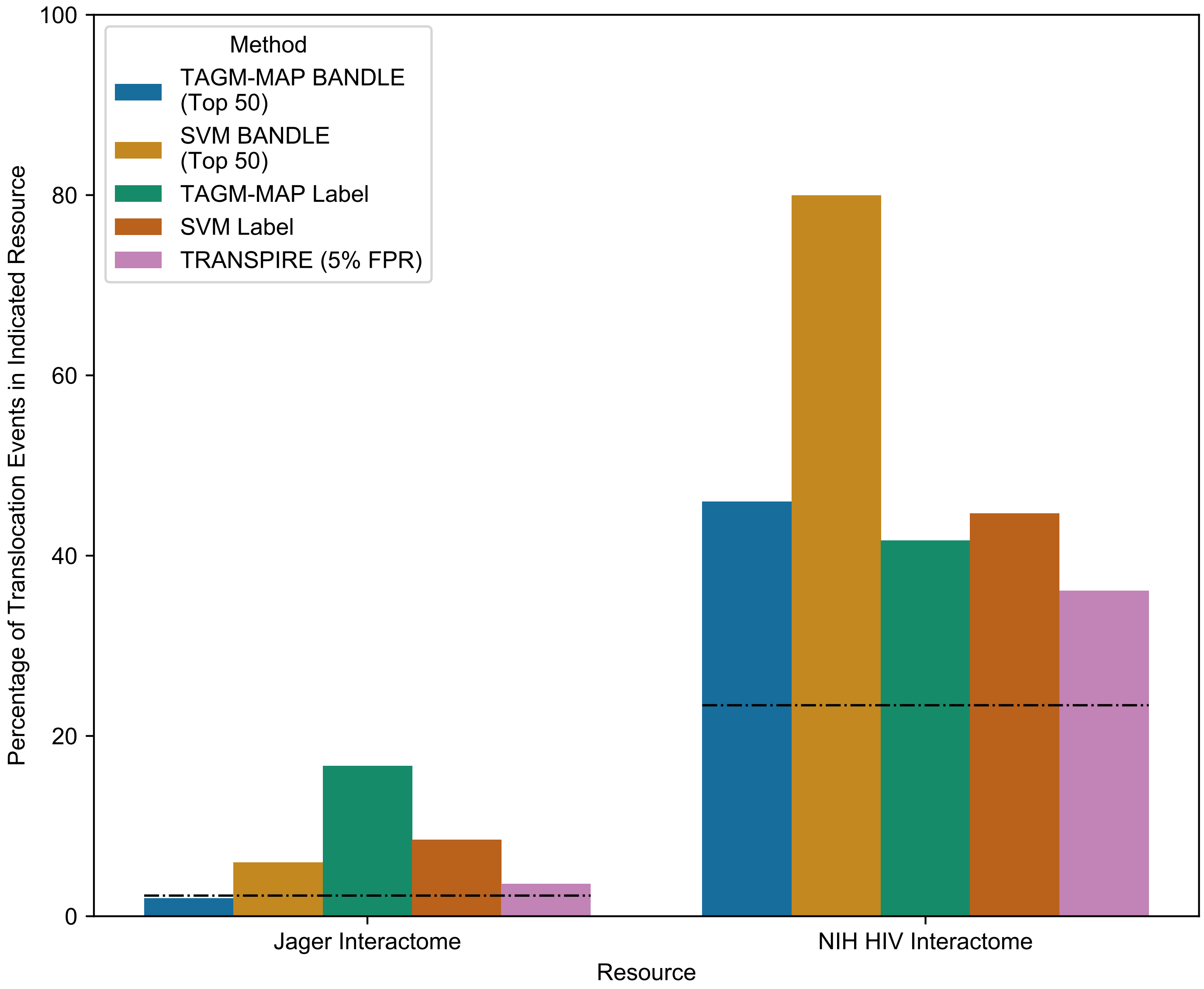

Method
TAGM-MAP BANDLE (Top 50)
SVM BANDLE (Top 50)
TAGM-MAP Label
SVM Label
TRANSPIRE (5% FPR)
Percentage of Translocation Events in Indicated Resource
0
20
40
60
80
100
Jager Interactome
NIH HIV Interactome
Resource

Supplemental Figure S11: Performance of translocation identification method depends on the choice of known HIV interactors in ΔNef replicates. The percentage of translocation events from each method that are in the Jäger HIV interactome[27] (left bars) or the NIH HIV interactome[28] (right bars) is shown. Dashed lines indicate the percentage of translocation events that would be expected by chance based on the proportion of the human proteome represented in each interactome.

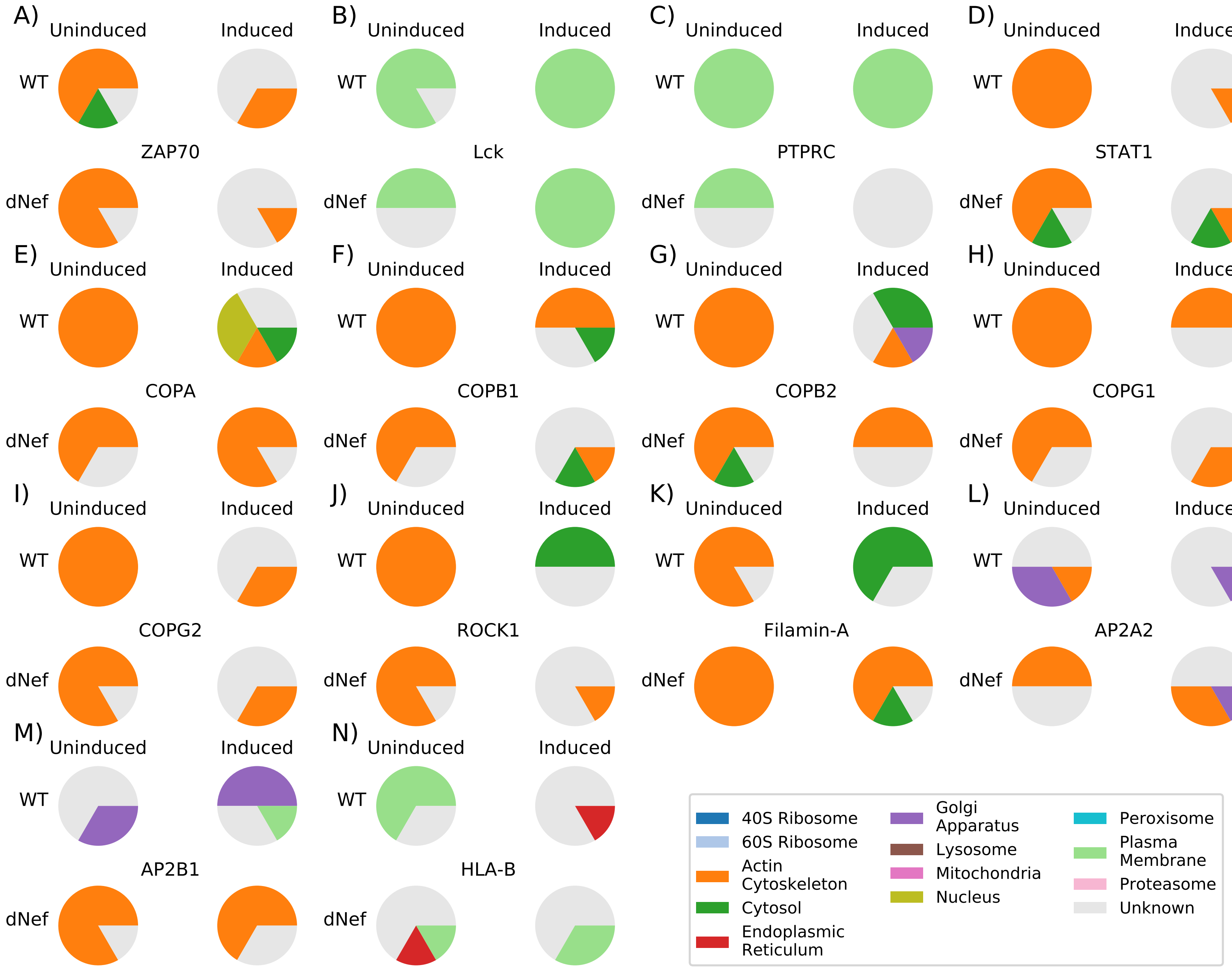
A)
Uninduced
Induced
WT
ZAP70
dNef
B)
Uninduced
Induced
WT
Lck
dNef
C)
Uninduced
Induced
WT
PTPRC
dNef
D)
Uninduced
Induced
WT
STAT1
dNef
E)
Uninduced
Induced
WT
COPA
dNef
F)
Uninduced
Induced
WT
COPB1
dNef
G)
Uninduced
Induced
WT
COPB2
dNef
H)
Uninduced
Induced
WT
COPG1
dNef
I)
Uninduced
Induced
WT
COPG2
dNef
J)
Uninduced
Induced
WT
ROCK1
dNef
K)
Uninduced
Induced
WT
Filamin-A
dNef
L)
Uninduced
Induced
WT
AP2A2
dNef
M)
Uninduced
Induced
WT
AP2B1
dNef
N)
Uninduced
Induced
WT
HLA-B
dNef
40S Ribosome
60S Ribosome
Actin Cytoskeleton
Cytosol
Endoplasmic Reticulum
Golgi Apparatus
Lysosome
Mitochondria
Nucleus
Peroxisome
Plasma Membrane
Proteasome
Unknown

Supplemental Figure S12: SVM classification of selected SVM-based BANDLE translocation events. Pie charts displaying the classification across WT and ΔNef replicates for the WT only translocation events: TCR signaling proteins (A-C), STAT1 (D), and COPI complex (E-I). Cytoskeletal organizers ROCK1 (J) and Filamin-A (K) are common to both WT and ΔNef replicates. AP2A2 (L), AP2B1 (M), and HLA-B (N) were ΔNef only translocation events.

Figure S13

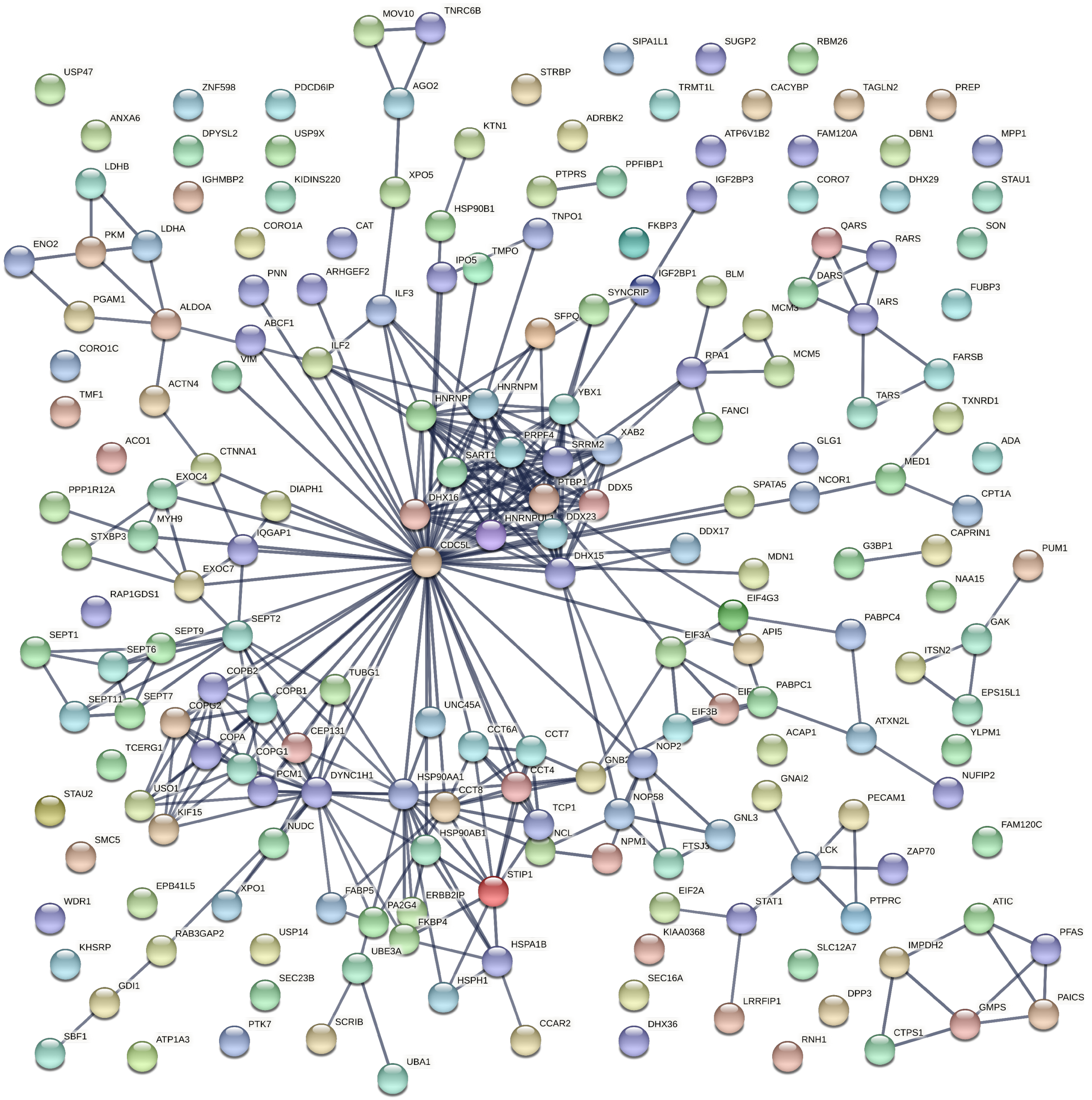

MOV10
TNRC6B
SIPA1L1
SUGP2
RBM26
USP47
ZNF598
PDCD6IP
AGO2
STRBP
TRMT1L
CACYBP
TAGLN2
PREP
ANXA6
DPYSL2
USP9X
KTN1
ADRBK2
ATP6V1B2
FAM120A
DBN1
MPP1
LDHB
IGHMBP2
KIDINS220
XPO5
PTPRS
PPFIBP1
IGF2BP3
CORO7
DHX29
STAU1
CORO1A
CAT
HSP90B1
TNPO1
FKBP3
QARS
SON
ENO2
PKM
LDHA
RARS
TMPO
IPO5
PNN
ARHGEF2
IGF2BP1
BLM
DARS
FUBP3
ILF3
SYNCRIP
IARS
PGAM1
ALDOA
ABCF1
SFPQ
MCM3
CORO1C
ILF2
RPA1
MCM5
FARSB
VIM
TMF1
ACTN4
HNRNPM
YBX1
TARS
TXNRD1
FANCI
XAB2
ACO1
PRPF4
SRRM2
GLG1
ADA
CTNNA1
SART1
MED1
EXOC4
PTBP1
DDX5
SPATA5
NCOR1
PPP1R12A
DIAPH1
DHX16
CPT1A
MYH9
DDX23
DDX17
STXBP3
IQGAP1
CDC5L
DHX15
CAPRIN1
G3BP1
MDN1
PUM1
EXOC7
NAA15
RAP1GDS1
EIF4G3
SEPT2
PABPC4
GAK
SEPT1
SEPT9
EIF3A
API5
SEPT6
ITSN2
COPB2
TUBG1
PABPC1
SEPT11
SEPT7
COPB1
EPS15L1
COPG2
UNC45A
EIF3B
ATXN2L
CEP131
CCT6A
COPA
CCT7
ACAP1
YLPM1
TCERG1
COPG1
NOP2
CCT4
PCM1
DYNC1H1
HSP90AA1
GNB2
GNAI2
NUFIP2
STAU2
USO1
CCT8
NOP58
PECAM1
KIF15
TCP1
GNL3
NUDC
HSP90AB1
NCL
NPM1
FAM120C
SMC5
FTSJ3
LCK
ZAP70
STIP1
EPB41L5
XPO1
FABP5
ERBB2IP
EIF2A
PA2G4
ATIC
WDR1
STAT1
PTPRC
FKBP4
RAB3GAP2
USP14
UBE3A
KIAA0368
PFAS
HSPA1B
IMPDH2
KHSRP
SLC12A7
SEC23B
SEC16A
GDI1
HSPH1
GMPS
PAICS
DPP3
LRRFIP1
SBF1
PTK7
SCRIB
CCAR2
DHX36
CTPS1
ATP1A3
RNH1
UBA1

Supplemental Figure S13: STRING diagram of SVM-based BANDLE translocation events found only for WT replicates. Network edges indicate confidence of protein connection at a minimum interaction score of 0.9.

Figure S14

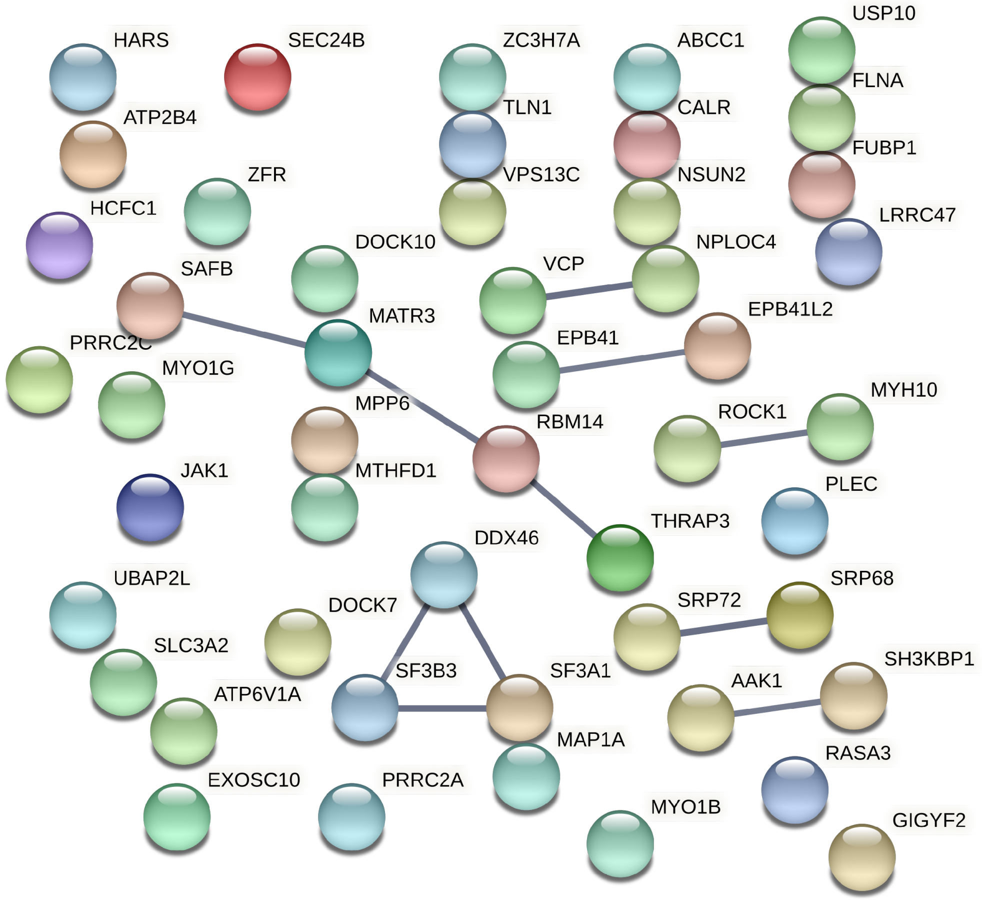

HARS
SEC24B
ZC3H7A
ABCC1
USP10
FLNA
ATP2B4
TLN1
CALR
FUBP1
ZFR
VPS13C
NSUN2
HCFC1
LRRC47
DOCK10
NPLOC4
SAFB
VCP
MATR3
EPB41L2
PRRC2C
EPB41
MYO1G
MYH10
MPP6
ROCK1
RBM14
MTHFD1
JAK1
PLEC
THRAP3
DDX46
UBAP2L
SRP68
DOCK7
SRP72
SLC3A2
SF3B3
SF3A1
SH3KBP1
AAK1
ATP6V1A
MAP1A
RASA3
EXOSC10
PRRC2A
MYO1B
GIGYF2

Supplemental Figure S14: STRING diagram of SVM-based BANDLE translocation events found for both WT and ΔNef replicates. Network edges indicate confidence of protein connection at a minimum interaction score of 0.9.

Figure S15

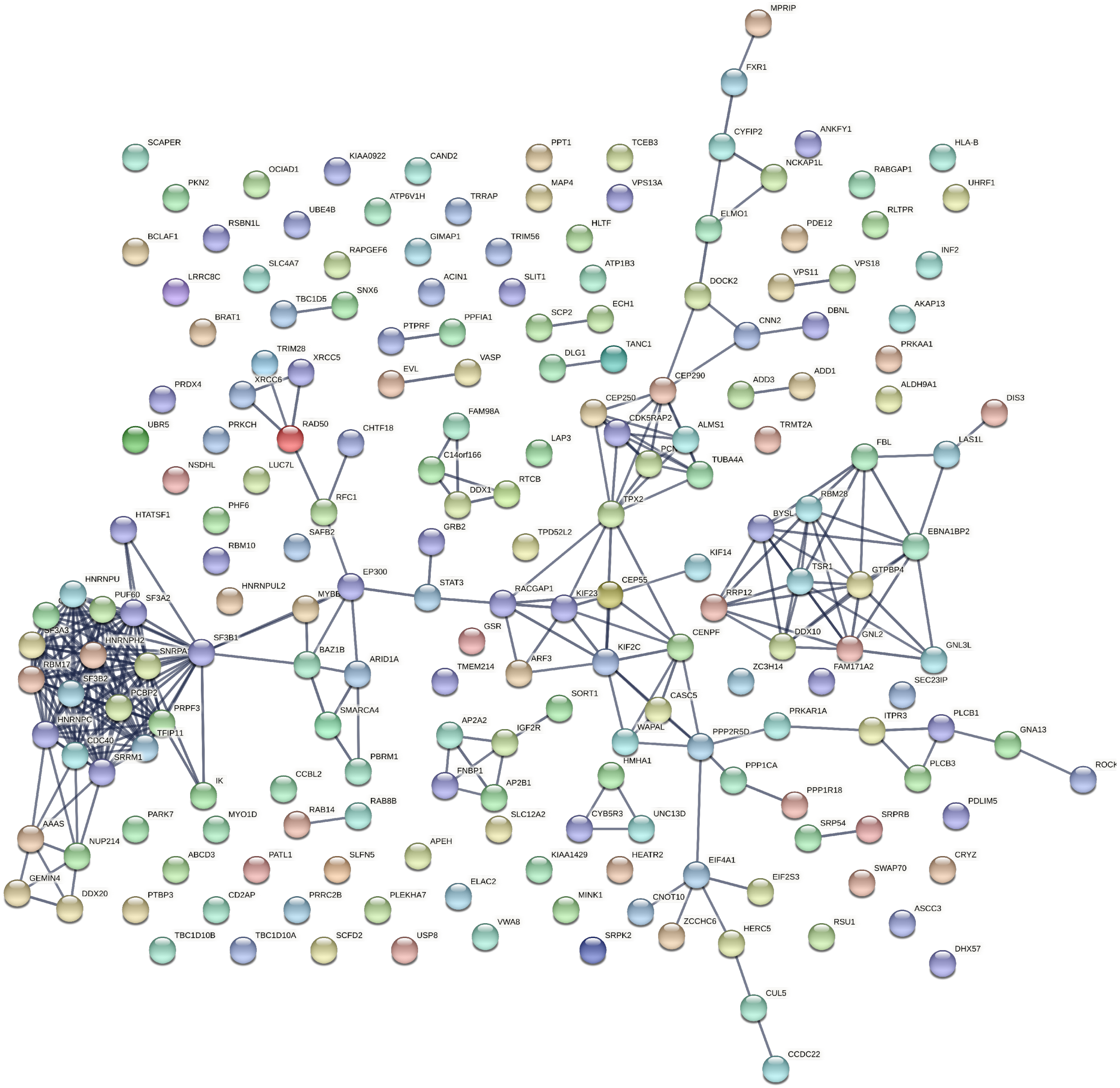
MPRIP
FXR1
CYFIP2
ANKFY1
NCKAP1L
ELMO1
DOCK2
CNN2
DBNL
CEP290
CEP250
CDK5RAP2
ALMS1
PCN
TUBA4A
TPX2
SCAPER
PKN2
OCIAD1
KIAA0922
CAND2
PPT1
TCEB3
HLA-B
RABGAP1
UHRF1
MAP4
VPS13A
UBE4B
RSBN1L
ATP6V1H
TRRAP
BCLAF1
GIMAP1
TRIM56
HLTF
PDE12
RLTPR
RAPGEF6
SLC4A7
LRRC8C
ACIN1
SLIT1
ATP1B3
VPS11
VPS18
INF2
TBC1D5
SNX6
BRAT1
PTPRF
PPFIA1
SCP2
ECH1
AKAP13
TRIM28
XRCC5
XRCC6
EVL
VASP
DLG1
TANC1
PRKAA1
PRDX4
ADD3
ADD1
ALDH9A1
DIS3
UBR5
PRKCH
RAD50
CHTF18
FAM98A
LAP3
TRMT2A
NSDHL
LUC7L
C14orf166
DDX1
RTCB
FBL
LAS1L
RBM28
PHF6
RFC1
SAFB2
BYSL
EBNA1BP2
HTATSF1
TPD52L2
GRB2
KIF14
RBM10
TSR1
GTPBP4
EP300
STAT3
RACGAP1
KIF23
CEP55
HNRNPU
PUF60
SF3A2
HNRNPUL2
MYBB
RRP12
SF3B1
SF3A3
HNRNPH2
SNRPA
GSR
CENPF
DDX10
GNL2
GNL3L
BAZ1B
KIF2C
RBM17
ARID1A
TMEM214
ARF3
ZC3H14
FAM171A2
SEC23IP
SF3B2
PCBP2
SORT1
CASC5
PRPF3
SMARCA4
AP2A2
IGF2R
PRKAR1A
ITPR3
PLCB1
GNA13
HNRNPC
TFIP11
WAPAL
PPP2R5D
CDC40
SRRM1
PBRM1
HMHA1
PPP1CA
PLCB3
ROCK
IK
CCBL2
FNBP1
AP2B1
PPP1R18
RAB14
RAB8B
PDLIM5
PARK7
MYO1D
SLC12A2
CYB5R3
UNC13D
SRP54
SRPRB
AAAS
NUP214
APEH
ABCD3
PATL1
SLFN5
KIAA1429
HEATR2
EIF4A1
SWAP70
CRYZ
GEMIN4
EIF2S3
DDX20
PTBP3
CD2AP
PRRC2B
PLEKHA7
ELAC2
MINK1
CNOT10
ZCCHC6
HERC5
RSU1
ASCC3
TBC1D10B
TBC1D10A
SCFD2
USP8
VWA8
SRPK2
DHX57
CUL5
CCDC22

<u>Supplemental Figure S15:</u> STRING diagram of SVM-based BANDLE translocation events found only for ΔNef replicates. Network edges indicate confidence of protein connection at a minimum interaction score of 0.9.
